# Pathogenic mutations reveal a role of RECQ4 in mitochondrial RNA:DNA hybrid formation and resolution

**DOI:** 10.1038/s41598-020-74095-9

**Published:** 2020-10-12

**Authors:** Chou-Wei Chang, Xiaohua Xu, Min Li, Di Xin, Lin Ding, Ya-Ting Wang, Yilun Liu

**Affiliations:** 1 Department of Cancer Genetics and Epigenetics, Beckman Research Institute, City of Hope, Duarte, CA 91010-3000 USA; 2 J. Craig Venter Institute, San Diego, CA 92037 USA; 3 Memorial Sloan Kettering, New York, NY 10065 USA

**Keywords:** Molecular biology, DNA replication, DNA synthesis, Mitochondria

## Abstract

The synthesis of mitochondrial DNA (mtDNA) is a complex process that involves the formation and resolution of unusual nucleic acid structures, such as RNA:DNA hybrids. However, little is known about the enzymes that regulate these processes. RECQ4 is a DNA replication factor important for mtDNA maintenance, and here, we unveil a role of human RECQ4 in regulating the formation and resolution of mitochondrial RNA:DNA hybrids. Mitochondrial membrane protein p32 can block mtDNA synthesis by restricting RECQ4 mitochondrial localization via protein–protein interaction. We found that the interaction with p32 was disrupted not only by the previously reported cancer-associated RECQ4 mutation, del(A420-A463), but also by a clinical mutation of the adjacent residue, P466L. Surprisingly, although P466L mutant was present in the mitochondria at greater levels, unlike del(A420-A463) mutant, it failed to enhance mtDNA synthesis due to the accumulation of RNA:DNA hybrids throughout the mtDNA. Biochemical analysis revealed that P466L mutation enhanced RECQ4 annealing activity to generate RNA:DNA hybrids at the same time reduced its unwinding activity to resolve this structure. Hence, P466L mutation led to a reduced efficiency in completing mtDNA synthesis due to unresolved RNA:DNA hybrids across mtDNA.

## Introduction

Mitochondria generate the ATP needed by diverse cellular processes to support cell growth. In recent years, mitochondria have gained attention for their potential use as diagnostic tools and as therapeutic targets for cancer treatment^1^. Mitochondrial DNA (mtDNA) is small circular DNA that resides in the mitochondria and encodes a subset of the genes required for oxidative phosphorylation (OXPHOS) function to generate ATP^2^. Although each cell may contain multiple copies of mtDNA^3^, aberrant mtDNA copy numbers in cancer cells have been correlated with tumor aggressiveness and poor survival outcome^1,4–11^. In addition, reduced mtDNA copy numbers have been linked to multiple diseases, including aging-associated neural/cognitive deterioration and Huntington’s disease^12,13^. Clearly, the impact of mtDNA copy number on human health is multi-fold.

mtDNA synthesis initiates from the heavy (H)-strand replication origin, O_H_, through the formation of an RNA:DNA hybrid, also known as an R-loop. The RNA:DNA hybrid that serves as a primer for DNA polymerase γ (pol γ) to synthesize H-strand DNA at O_H_ is generated by a transcriptional event initiated at the light-strand promoter (LSP)^14^. To initiate replication, transcription must first be terminated to allow the switch to replication^15^. RNA:DNA hybrid not only is required for the replication initiation step at O_H_, but this structure also forms extensively as replication intermediates throughout the mtDNA during mtDNA synthesis. This is because even though similar to nuclear DNA replication, mtDNA can be synthesized via strand-coupled, bi-directional mechanism^16^, in human cell lines, mtDNA synthesis is primarily asymmetric, as L-strand synthesis does not initiate until H-strand synthesis reaches the L-strand replication origin, O_L_, approximately 2/3 of the way around the molecule^14,17^. During H-strand synthesis, the displaced single-stranded H-strand template is protected either by the binding of mitochondrial single-stranded binding protein (mtSSB) or by extensive RNA:DNA hybrid formation through a phenomenon known as RNA-incorporation-throughout-the-lagging-strand (RITOLS)^14,18^. For L-strand synthesis to take place, the bound mtSSB or RNA:DNA hybrids must be removed from the H-strand template. Although the formation and resolution of these extensive RNA:DNA hybrids across mtDNA during mtDNA synthesis have been observed in cells^14^, we have little knowledge of the enzymes involved in these complex processes.

RECQ4, a member of the highly conserved RECQ family of superfamily 2 (SF2) DNA helicases^19^, is a critical enzyme for embryonic development^20^. RECQ4 mutations have been identified in patients suffering from Rothmund-Thomson syndrome (RTS), Baller-Gerold syndrome (BGS), and RAPADILINO syndrome, with phenotypes ranging from developmental defects to premature aging and cancer predisposition^21^. In addition to the SF2 helicase domain, RECQ4 contains a unique N-terminus that resembles the essential yeast replication initiation factor Sld2^22^. In human cells, RECQ4 is a key component of both the nuclear and mitochondrial replication helicase complexes^23,24^. In the nucleus, RECQ4 is important for DNA replication initiation because it promotes the assembly of the active CDC45-MCM2-7-GINS replicative helicase complex^22,23,25–27^. In mitochondria, RECQ4 interacts with the mitochondrial replication helicase TWINKLE and is crucial for maintaining mtDNA copy number in human cells^24,28,29^. Previously, we purified RECQ4 protein complexes from various cellular compartments and identified additional RECQ4-interacting proteins, including p32, which directly interacts with RECQ4 and negatively regulates its mitochondrial localization^24^. This regulation is important for controlling the rate of mtDNA synthesis. One of the most common clinical RECQ4 mutations (1390 + 2delT) results in an internal deletion (ID) between residues A420 and A463^30^. Cells expressing RECQ4 ID mutant abolished RECQ4-p32 interaction and exhibited increased mitochondrial accumulation of RECQ4 and mtDNA synthesis rate^24^.

Although the involvement of RECQ4 in mtDNA copy number maintenance has been established, the mechanism by which RECQ4 contributes to mtDNA synthesis remains elusive. In the present study, we show that RECQ4 is important for the formation and resolution of RNA:DNA hybrid both at the replication origin O_H_ to initiate DNA synthesis and as replication intermediates throughout the mtDNA to facilitate RITOLS. We present evidence that the function of RECQ4 in regulating RNA:DNA hybrids on mtDNA was deregulated by clinical mutation, P466L^30–32^. P466L mutant exhibited higher affinity for mtDNA in cells, but mtDNA bound by the P466L mutant accumulated unresolved RNA:DNA hybrids compared to mtDNA bound by wild-type (WT) or the RECQ4 ID mutant. In vitro biochemical analyses demonstrated that RECQ4 P466L mutant exhibited enhanced annealing activity to generate RNA:DNA hybrids but reduced unwinding activity to resolve the same structure. Although RNA:DNA hybrids serve to stabilize the H-strand^14,18^, the persistent presence of RNA:DNA hybrids can hinder the initiation and progression of second-strand DNA synthesis, leading to reduced mtDNA copy number and altered mitochondrial respiratory efficiency in RECQ4 P466L mutant cells.

## Results

### RECQ4 is important for the initiation of mtDNA synthesis

**Figure 1:**
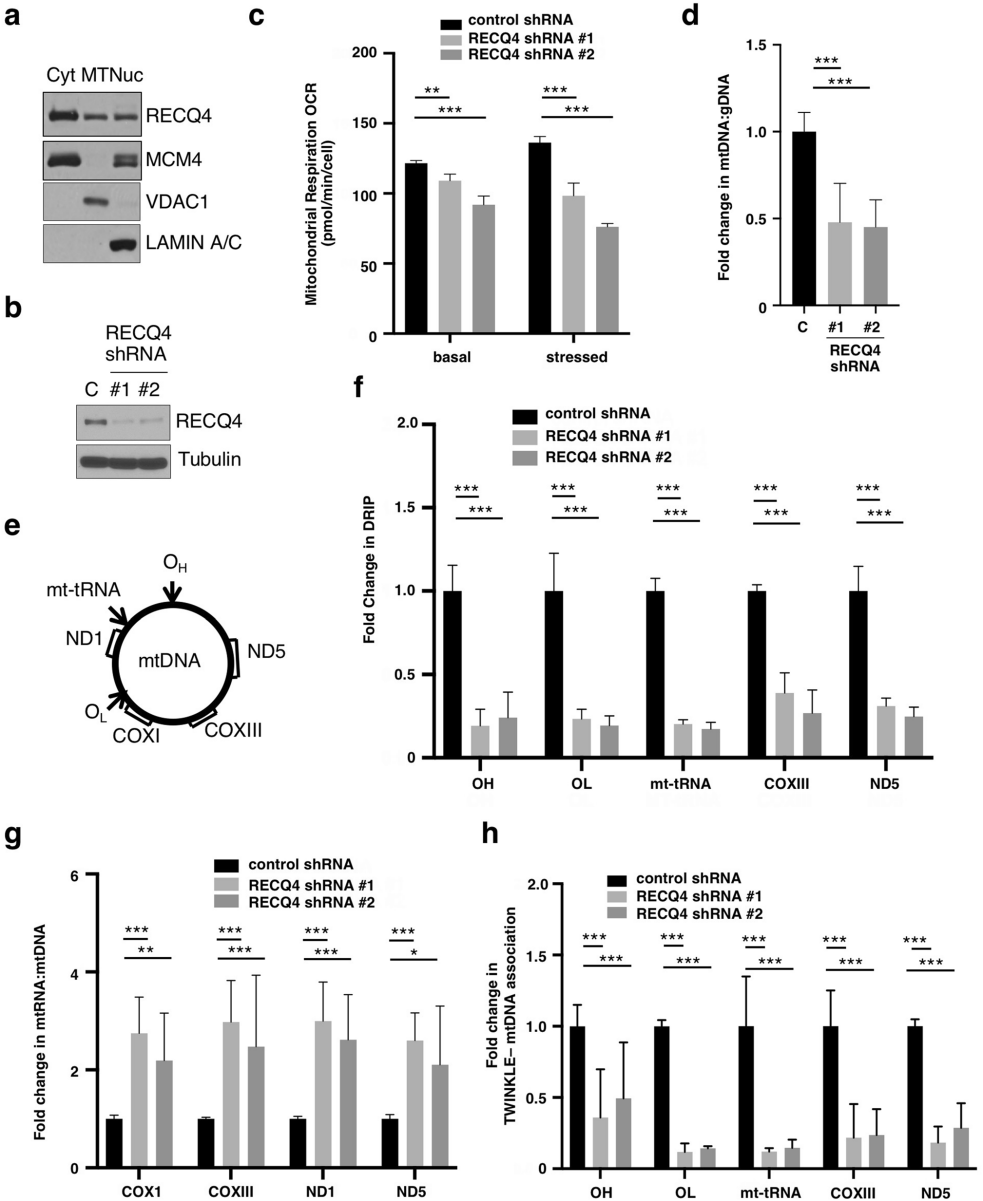
RECQ4 is important for the initiation of mtDNA synthesis. (**a**) Western blot analysis of the indicated proteins in cytosolic (Cyt), mitochondrial (MT), and nuclear (Nuc) fractions prepared from HEK293 cells. VDAC1 and lamin A/C are loading and fractionation controls for MT and Nuc fractions, respectively. (**b**) Western blot analysis of RECQ4 protein expression in whole cell extracts (WCEs) prepared from doxycycline-treated HEK293 cells containing inducible control or RECQ4 shRNA. Tubulin is used as a loading control. (**c**) Analysis of the oxygen consumption rate (OCR) measured under basal (left) and stressed (right) conditions using a Seahorse Analyzer to assess mitochondrial function in control and RECQ4-depleted HEK293 cells. The Y-axis shows OCR values normalized to cell numbers measured by MTT assays. ** indicates *p* value < 0.01, and *** indicates *p* value < 0.005. (**d**) Fold changes in mtDNA to genomic DNA (gDNA) ratio in RECQ4-depleted HEK293 cells relative to control shRNA cells. (**e**) Schematic diagram of mtDNA indicating the locations of the replication origins and genes that were analyzed in this study. (**f**) Fold changes in RNA:DNA hybrid frequency at O_H_ (OH), O_L_ (OL), COXIII, ND5 and mt-tRNA loci on mtDNA in RECQ4 shRNA-expressing HEK293 cells relative to the control shRNA cells. The input mtDNA were treated with or without RNase H prior to S9.6 immunoprecipitation. Immunopurified mtDNA sequence was measured using qPCR and normalized to the qPCR quantification of the input mtDNA minus the quantifications from the RNase H treatment. (**g**) Fold changes in the indicated mitochondrial RNA (mtRNA) transcripts measured by reverse-transcription-coupled quantitative PCR (RT-qPCR) relative to mtDNA levels (mtRNA:mtDNA) in RECQ4 shRNA-expressing HEK293 cells as compared to control shRNA cells. (**h**) Fold changes in the amount of mtDNA detected by qPCR that was co-purified with TWINKLE after normalized with the total mtDNA input prior to immunoprecipitation in RECQ4-depleted HEK293 cells as compared to control shRNA cells. All quantitative analyses shown in Fig. 1 are based on mean ± standard deviation of 3 independent biological experiments, each with 3 triplicate reactions.

RECQ4 localized to multiple cellular compartments, including the mitochondria (Fig. 1a)^24,29,33^. Using Seahorse mitochondrial stress tests to measure mitochondrial oxygen consumption rate (OCR), we found that HEK293 cells containing doxycycline (DOX)—inducible shRNA specific to RECQ4 (Fig. 1b) exhibited reduced basal mitochondrial respiratory OCR than cells containing control-shRNA (Fig. 1c, left). Mitochondrial respiratory OCR remained at significantly lower rates in RECQ4-depleted cells than in control cells after treatment with carbonyl cyanmide-p-trifluoromethox-yphenyl-hydrazon (FCCP) to induce maximum electron transport chain function (i.e. stressed condition; Fig. 1c, right), indicating that OXPHOS was reduced after RECQ4 depletion. On the other hand, RECQ4 depletion did not significantly alter the rate of glycolysis, as measured by the extracellular acidification rate (ECAR) under both basal and stressed conditions (Supplementary Fig. 1a).

The reduced mitochondrial OCR correlates with the lower levels of mtDNA in RECQ4-depleted cells as compared to control shRNA-treated cells (Fig. 1d). Consistent with a role of RECQ4 in mtDNA synthesis^24,34^, we found that RECQ4-depleted cells exhibited a slower mtDNA recovery compared to cells containing control shRNA after mtDNA was depleted by ethidium bromide (EtBr; Supplementary Fig. 1b,c). We next determined if the defect in mtDNA synthesis in the absence of RECQ4 occurred at the initiation step. For this, we measured the formation of RNA:DNA hybrids at O_H_ (Fig. 1e) by DNA:RNA immunoprecipitation (DRIP) using an anti-S9.6 antibody, followed by quantitative PCR (qPCR) analysis, and found that RNA:DNA hybrids at O_H_ were indeed significantly reduced in HEK293 cells after RECQ4 shRNA inductions (Fig. 1f). RNA:DNA hybrids not only serve as RNA primers for initiating DNA synthesis at O_H_, but they are also present extensively at other regions of the mtDNA as replication intermediates during asymmetric mtDNA synthesis^14,18^. Consistent with a defect in replication initiation at O_H_, the amount of RNA:DNA hybrids as replication intermediates at non-O_H_ loci, such as replication origin O_L_, *COXIII*, *ND5* and mitochondrial transfer RNA (mt-tRNA) coding regions (Fig. 1e), also decreased after RECQ4 depletion induced by DOX treatment (Fig. 1f). It has been reported that the RNA molecules needed for generating RNA:DNA hybrids are transcribed in an abundant amount in the mitochondria, and these preformed RNAs can then anneal to the H-strand template in a 3′–5′ direction to protect the H-strand template during mtDNA synthesis^35^. We found that RECQ4-depleted cells generated more mitochondrial RNA (mtRNA) transcripts produced per mtDNA than control shRNA-treated cells (Fig. 1g), indicating that the reduced RNA:DNA hybrid formation after RECQ4 depletion was not due to decreased mtRNA transcripts.

The lack of RNA priming at O_H_ in RECQ4-depleted cells suggested a defect in the subsequent step in recruiting mitochondrial replication factors, such TWINKLE replicative helicase, to mtDNA. To test this, we fixed protein-DNA complexes in control and RECQ4 shRNA-treated HEK293 cells and then purified TWINKLE-DNA complexes using an anti-TWINKLE antibody. We subjected the co-purified DNA fragments to qPCR analysis for the presence of mtDNA. Indeed, after normalized to the input mtDNA, we found that TWINKLE associations with all mitochondrial regions, including O_H_, were reduced in cells after RECQ4 shRNA induction (Fig. 1h). Altogether, our analysis supports a role of RECQ4 in facilitating the formation of RNA:DNA hybrid to promote the initiation of mtDNA synthesis.

### Clinical P466L mutation promotes RECQ4 mitochondrial accumulation

**Figure 2:**
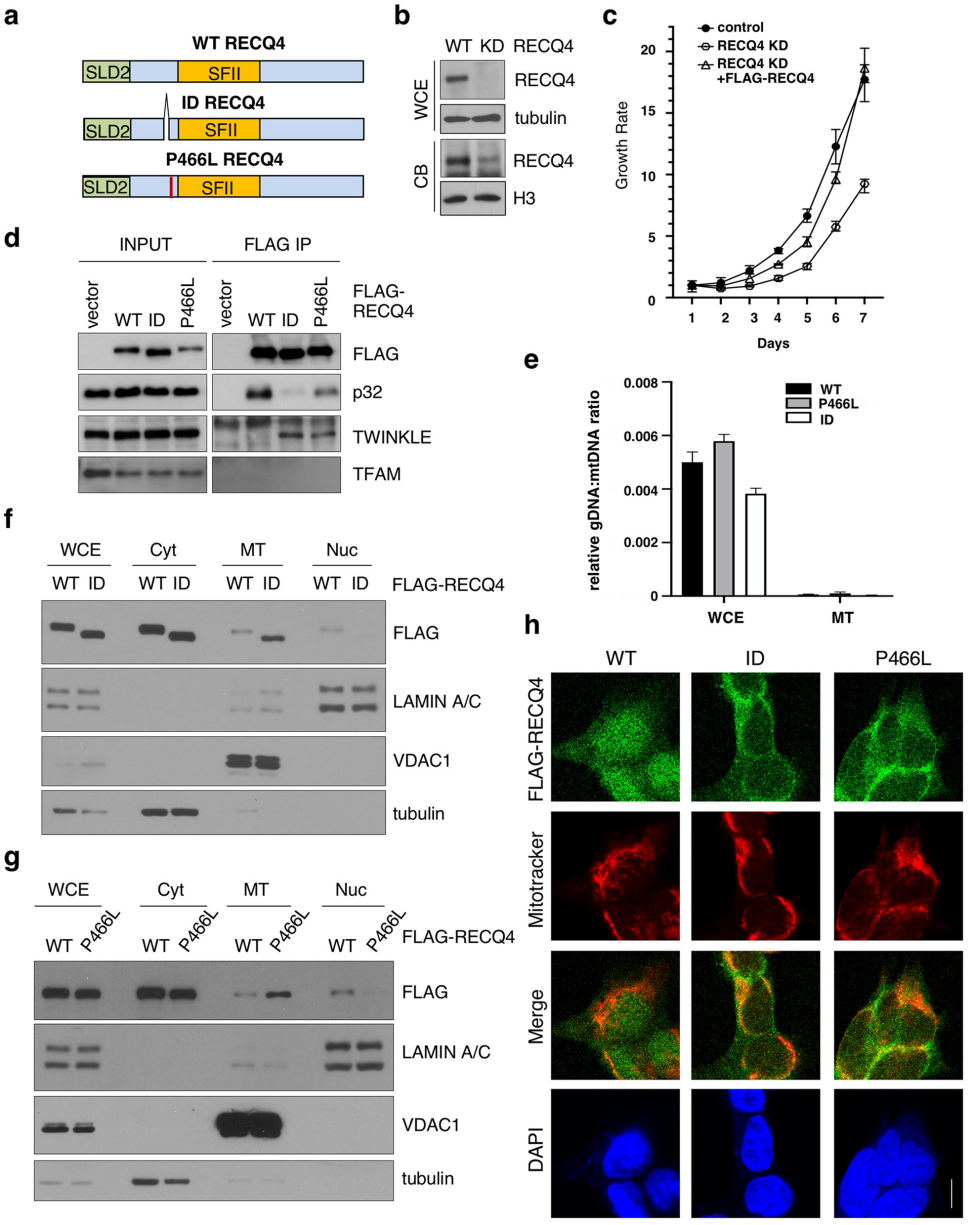
The P466L clinical mutation leads to RECQ4 mitochondrial accumulation. (**a**) Schematic of human RECQ4 WT, ID and P466L mutant proteins, including the SLD2 (green) and conserved SF2 helicase domains (yellow). (**b**) Western blot analysis of RECQ4 in WCEs and chromatin-bound (CB) fractions prepared from HEK293 WT or RECQ4 knockdown (KD) HEK293 cells generated by CRISPR technology. Tubulin is used as a loading control. (**c**) The effects of stable RECQ4 KD shown in (**b**) and complementation using FLAG-RECQ4 on cell growth as measured by crystal violet cell proliferation assays. Each value represents mean ± standard deviation of 3 independent biological experiments, each with 3 triplicate reactions. (**d**) Western blot analysis for the presence of WT and mutant FLAG-RECQ4, p32, TWINKLE and TFAM in WCE (left) and immunoprecipitated (IP) with FLAG-RECQ4 complexes (right) in WCEs prepared from stable RECQ4 KD HEK293 cells expressing FLAG-RECQ4 WT, ID or P466L mutant. (**e**) gDNA levels relative to mtDNA in WCE (left) and MT (right) prepared from stable RECQ4 KD HEK293 cells expressing FLAG-RECQ4 WT, ID or P466L mutant. (**f**) Western blot analysis of stable RECQ4 KD HEK293 cells expressing FLAG-RECQ4 WT or ID mutant in WCEs and Cyt, MT, and fractions. Tubulin, VDAC1, and lamin A/C are loading and fractionation controls for Cyt, MT, and Nuc fractions, respectively. (**g**) Western blot analysis of RECQ4 in WCEs and Cyt, MT, and Nuc fractions prepared from stable RECQ4 KD HEK293 cells expressing FLAG-RECQ4 WT or P466L mutant. (**h**) Representative images showing immunofluorescent staining of FLAG-RECQ4 (green) in stable RECQ4 KD HEK293 cells expressing indicated WT and mutant FLAG-RECQ4 proteins. Mitotracker (red) was used to detect mitochondria, and DAPI (blue) was used to detect nuclei.

To date, more than 50 mutations in the human *RECQ4* gene have been linked to RTS, BGS, and/or RAPADILINO^30^. One of these clinical mutations, P466L, is in proximity to the 44 amino acids (A420-A463) missing in the ID mutation (Fig. 2a). Because the ID mutant lacks the p32-interacting motif and is able to accumulate in the mitochondria^24^, we tested if P466L mutation has similar effect on RECQ4 mitochondrial localization. For this, we first attempted to generate stable RECQ4-deficient HEK293 cells by deleting the endogenous *RECQ4* alleles using CRISPR technology. Because RECQ4 is an essential protein^20^, we failed to isolate viable clones with large deletions to completely eliminate both *RECQ4* alleles. Nonetheless, CRISPR technology frequently generates frameshift mutations that yield viable cells with residual protein expression, or knockdown (KD)^36^. Therefore, we were able to isolate a HEK293 clone that contained RECQ4 frameshift mutations (Supplementary Fig. 2a–c), allowing a small but detectable amount of the RECQ4 proteins concentrated in the chromatin-bound (CB) fraction, even though more than 90% of the RECQ4 proteins was depleted in the whole cell extracts (WCE; Fig. 2b). These cells with reduced RECQ4 expression, or RECQ4 KD cells, exhibited slower growth rates, which were restored to a comparable level as the control HEK293 cells after the stable integration of FLAG-tagged RECQ4 construct into the RECQ4 KD cells (Fig. 2c). In addition to introducing the wildtype (WT) RECQ4 protein, we also generated RECQ4 KD cells stably expressing FLAG-RECQ4 ID or P466L mutant at similar levels (Fig. 2d). Like the ID mutant^24^, we found that P466L mutation also reduced RECQ4-p32 interaction and enhanced RECQ4 interaction with the TWINKLE helicase (Fig. 2d), suggesting that P466L mutant proteins also accumulate in mitochondria. To test this possibility, we fractionated RECQ4 HEK293 KD cells or U2OS KD cells (Supplementary Fig. 2d–h) stably expressing FLAG-RECQ4 ID or P466L mutant proteins to obtain cytosolic (Cyt), mitochondrial (MT) and nuclear (Nuc) fractions, as previously described^24,37^. We further confirmed minimum contamination of genomic DNA (gDNA) relative to mtDNA in the purified MT fractions (Fig. 2e). Western blot analysis also showed that the purified MT fractions were free of the proteins known to only localize to the cytosol (e.g. tubulin) or the nucleus (e.g. Lamin A/C) nuclear proteins (Fig. 2f,g). Using this fractionation method, we found that similar to the ID mutant cells (Fig. 2f), there were higher levels of RECQ4 P466L mutant proteins in the mitochondrial fraction compared to RECQ4 protein levels in the respective WT cells (Fig. 2g, Supplementary Fig. 3a). Immunofluorescent microscopy also confirmed that the amount of P466L mutant proteins co-localized with the mitochondria increased (Fig. 2h). These results indicate that like the ID mutant protein, P466L mutant protein accumulates in the mitochondria, a phenomenon consistent with a reduced interaction with p32.

### Mitochondrial RNA:DNA hybrids accumulate in RECQ4 P466L mutant cells

**Figure 3:**
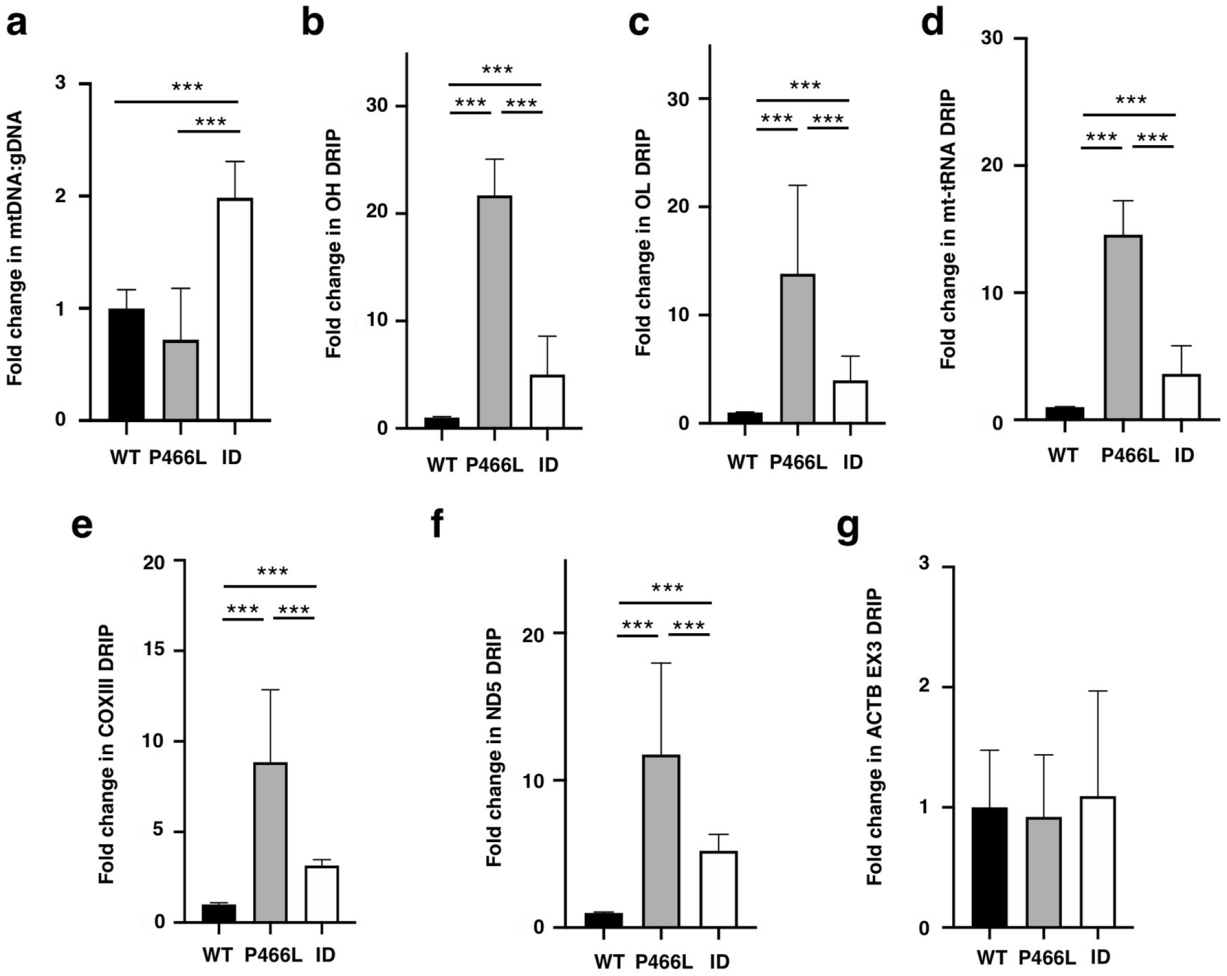
RNA:DNA hybrids accumulate on mtDNA in cells expressing P466L mutant RECQ4 proteins. (**a**) Fold changes in relative mtDNA:gDNA ratio in RECQ4 KD HEK293 cells expressing P466L or ID mutant RECQ4 as compared to cells expressing WT RECQ4. *** indicates *p* value < 0.005. (**b**–**f**) Fold changes in RNA:DNA hybrid frequency at (**b**) O_H_ (OH), (**c**) O_L_ (OL), (**d**) mt-tRNA, (**e**) COXIII and (**f**) ND5 gene loci on mtDNA in stable RECQ4 KD HEK293 cells expressing FLAG-RECQ4 P466L or ID mutant constructs as compared to cells expressing WT RECQ4. RNA:DNA hybrid frequency was measured by DRIP as described in Fig. 1f. (**h**) Fold changes in RNA:DNA hybrid frequency at exon 3 of *ACTB* (Ex3) in RECQ4 KD HEK293 cells expressing P466L FLAG-RECQ4 constructs compared to RECQ4 KD HEK293 cells expressing the WT proteins were analyzed by DRIP. For all quantitative analyses shown in Fig. 3, each value represents mean ± standard deviation of 3 independent biological experiments, each with 3 triplicate reactions.

We expected that the elevated RECQ4 P466L mutant proteins in the mitochondria also enhance mtDNA synthesis and increase mtDNA to gDNA ratio. Surprisingly, unlike ID mutant-expressing cells, P466L mutant-expressing HEK293 and U2OS cells did not enhance mtDNA levels (Fig. 3a; Supplementary Fig. 3b). After EtBr treatment to deplete mtDNA in both WT and P466L mutant cells (Supplementary Fig. 3c), we found that RECQ4 P466L mutant HEK293 cells showed slower mtDNA recovery as compared to cells expressing WT RECQ4 protein (Supplementary Fig. 3d). The inability of the elevated P466L mutant protein in the mitochondria to increase mtDNA production led us to determine if mtDNA replication initiation is defective in the P466L mutant cells by examining the formation of RNA:DNAs at O_H_. Surprisingly, we found that HEK293 cells expressing the P466L mutant exhibited a significant increase in RNA:DNA hybrids at O_H_, as compared to the WT or ID mutant cells (Fig. 3b). The increase in RNA:DNA hybrids was also observed at other regions of the mtDNA, such as O_L_ (Fig. 3c) and the non-origin loci (Fig. 3d–f). Importantly, this increase in P466L mutant cells was specific to mtDNA, as RNA:DNA hybrid levels in gDNA—including exon 3 of ACTB, which we have previously shown to contain RNA:DNA hybrids^38^—remained comparable between WT and P466L mutant cells (Fig. 3g). To lesser extent, we also observed accumulation of RNA:DNA hybrids on mtDNA in U2OS KD cells expressing the P466L mutant as compared to U2OS KD cells expressing either RECQ4 WT or ID mutant proteins (Supplementary Fig. 4a–d). These results together indicate that the accumulation of RNA:DNA hybrids at O_H_ is not due to a failure for the replication fork to progress beyond the initiation site, as RNA:DNA hybrids are formed as replication intermediates and further accumulate throughout the mtDNA.

**Figure 4:**
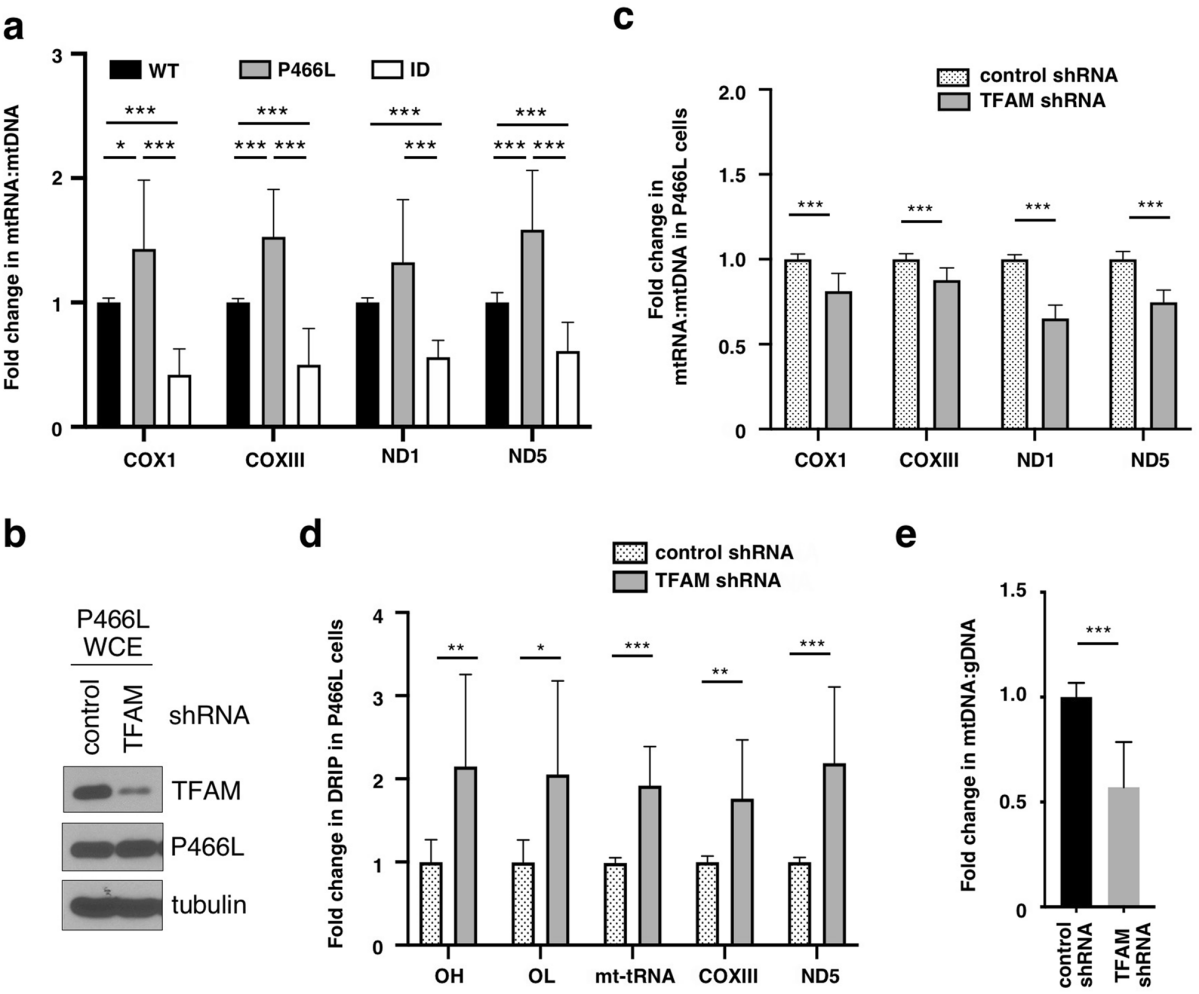
TFAM depletion does not reduce RNA:DNA hybrid accumulation on mtDNA in RECQ4 P466L mutant cells. (**a**) Fold changes in the mtRNA:mtDNA ratio in RECQ4 KD HEK293 cells expressing FLAG-RECQ4 P466L or ID constructs as compared to cells expressing FLAG-RECQ4 WT proteins. (**b**) Western blot analysis of TFAM protein expression in WCEs prepared from HEK293 KD cells expressing P466L mutant and treated with control or TFAM shRNA. Tubulin is used as a loading control. FLAG is used to monitor the expression of P466L mutant RECQ4. (**c**–**e**) Fold changes in (**c**) mtRNA:mtDNA ratio, (**d**) RNA:DNA hybrid frequency normalized to input mtDNA at the indicated loci and (**e**) relative mtDNA:gDNA ratio in HEK293 KD cells expressing P466L mutant and treated with TFAM shRNA as compared to P466L mutant cells treated with control shRNA as shown in (**b**). All quantitative analyses shown in Fig. 4 are based on mean ± standard deviation of 3 independent biological experiments, each with 3 triplicate reactions.

We next asked if the high levels of RNA:DNA hybrids were associated with altered mtRNA transcripts in the RECQ4 P466L mutant cells. Indeed, RECQ4 P466L mutant cells contained elevated levels of mtRNA transcripts produced per mtDNA compared to WT cells (Fig. 4a). However, when we depleted transcription factor TFAM using inducible TFAM-specific shRNA (Fig. 4b) to reduce mtRNA production in P466L mutant cells (Fig. 4c), we found that the amount of RNA:DNA hybrids in all loci tested further increased in RECQ4 P466L mutant cells after the induction of TFAM shRNA (Fig. 4d). The further increase in RNA:DNA hybrid levels after TFAM depletion also led to reduced mtDNA levels in the RECQ4 P466L mutant cells compared to those treated with control shRNA (Fig. 4e). Taken together, these results indicate that increased RNA:DNA hybrid formation in RECQ4 P466L mutant cells is not a consequence of the elevated levels of mtRNA, and that TFAM also has a role in regulating the level of RNA:DNA hybrids on mtDNA that is non-redundant to the role of RECQ4. We infer that the significant accumulation of RNA:DNA hybrids on mtDNA isolated from RECQ4 P466L mutant cells hinder the completion of mtDNA synthesis in these cells, which explains why the elevated level of P466L mutant proteins in the mitochondria fails to enhance mtDNA production in the mutant cells.

### RNA:DNA annealing but reduces RNA:DNA unwinding by RECQ4

**Figure 5:**
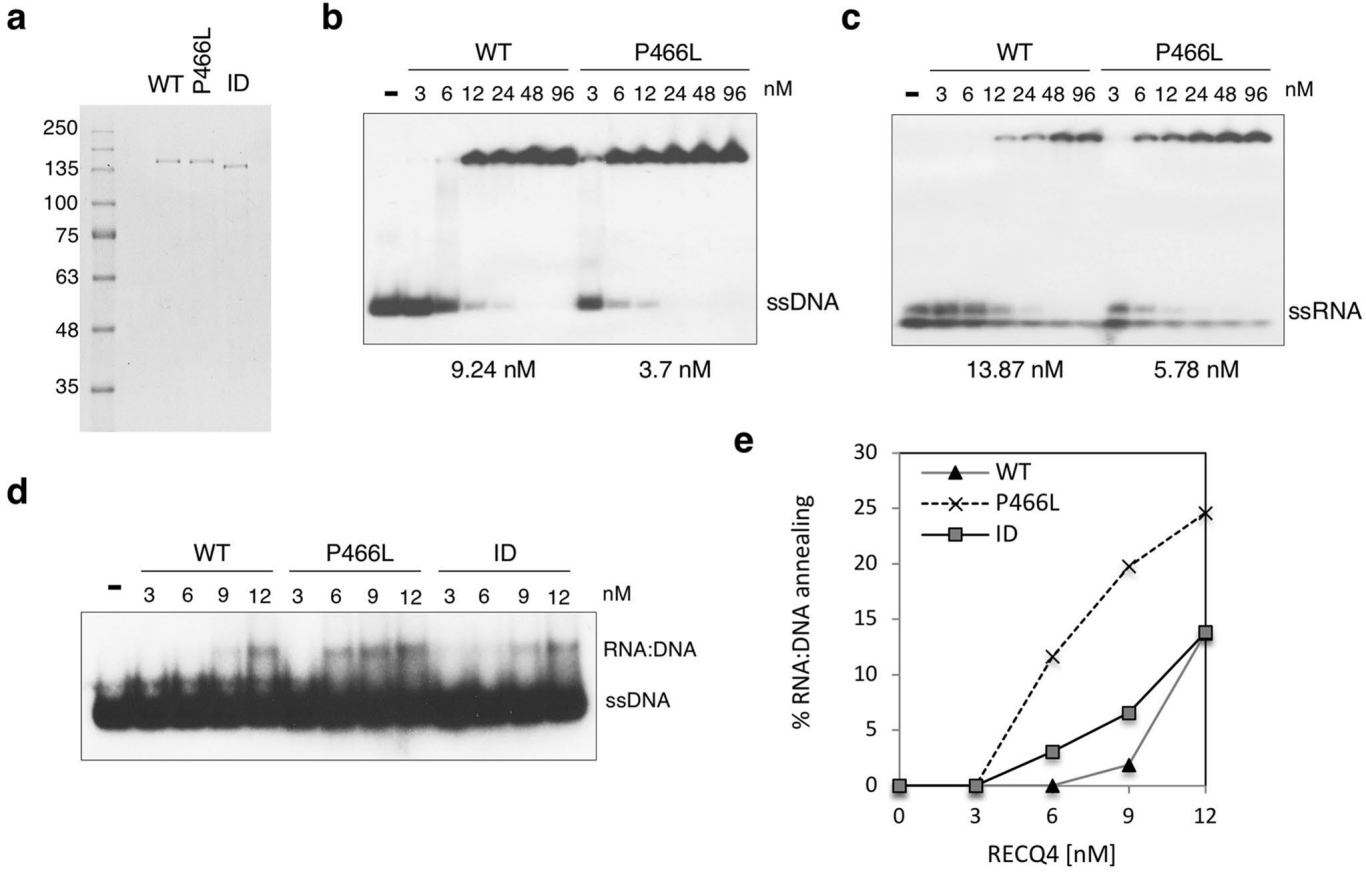
P466L mutant RECQ4 proteins exhibit higher nucleic acid binding and RNA:DNA annealing activity. (**a**) Recombinant WT, P466L and ID mutant RECQ4 proteins purified from *E. coli*, separated by SDS-PAGE, and stained with Coomassie blue. (**b**,**c**) Analysis of binding activity of purified recombinant RECQ4 WT and P466L mutant proteins shown in (**a**) using (**b**) ^32^P-end labeled single-stranded DNA (ssDNA) or (**c**) single-stranded RNA (ssRNA) on 5% native polyacrylamide gels. Binding affinities (K_a_) indicated below the gel were quantified using ImageJ. (**d**) Analysis of RNA:DNA annealing activity of purified recombinant WT, P466L and ID mutant RECQ4 proteins measured using ^32^P-end labeled X01 ssDNA with partial complementary RNA oligonucleotides. (**e**) The percentages of ^32^P-end labeled X01 forming RNA:DNA hybrids shown in (**d**) were quantified using ImageJ software.

We next wished to determine the mechanism by which RECQ4 P466L mutation accumulates RNA:DNA hybrids on mtDNA. Since RNA:DNA hybrids were generated throughout the H-strand template during mtDNA synthesis by incorporating processed mtRNA transcripts onto the mtDNA^35^, an enzyme with RNA:DNA annealing activity is likely involved in this process. Equally, an enzyme with RNA:DNA unwinding activity is expected to resolve these replication intermediates to allow second strand mtDNA synthesis. We previously showed that RECQ4 contains both DNA annealing and unwinding activities^39^. Therefore, we hypothesized that RECQ4 is involved in either the formation or resolution of RNA:DNA hybrids on mtDNA. To test this hypothesis, we purified recombinant RECQ4 P466L mutant proteins from *E. coli* (Fig. 5a) and asked if P466L mutant enhances RNA:DNA hybrid formation via its annealing activity, which requires binding to single-stranded DNA (ssDNA) and single-stranded RNA (ssRNA) first. We found that the affinities of RECQ4 P466L mutant proteins to both ssDNA (Fig. 5b) and ssRNA (Fig. 5c) increased by more than twofold compared to the WT proteins. The enhancement correlates with higher efficiency in promoting annealing to generate RNA:DNA hybrids by the P466L mutant compared to RECQ4 WT and the ID mutant (Fig. 5d,e).

**Figure 6:**
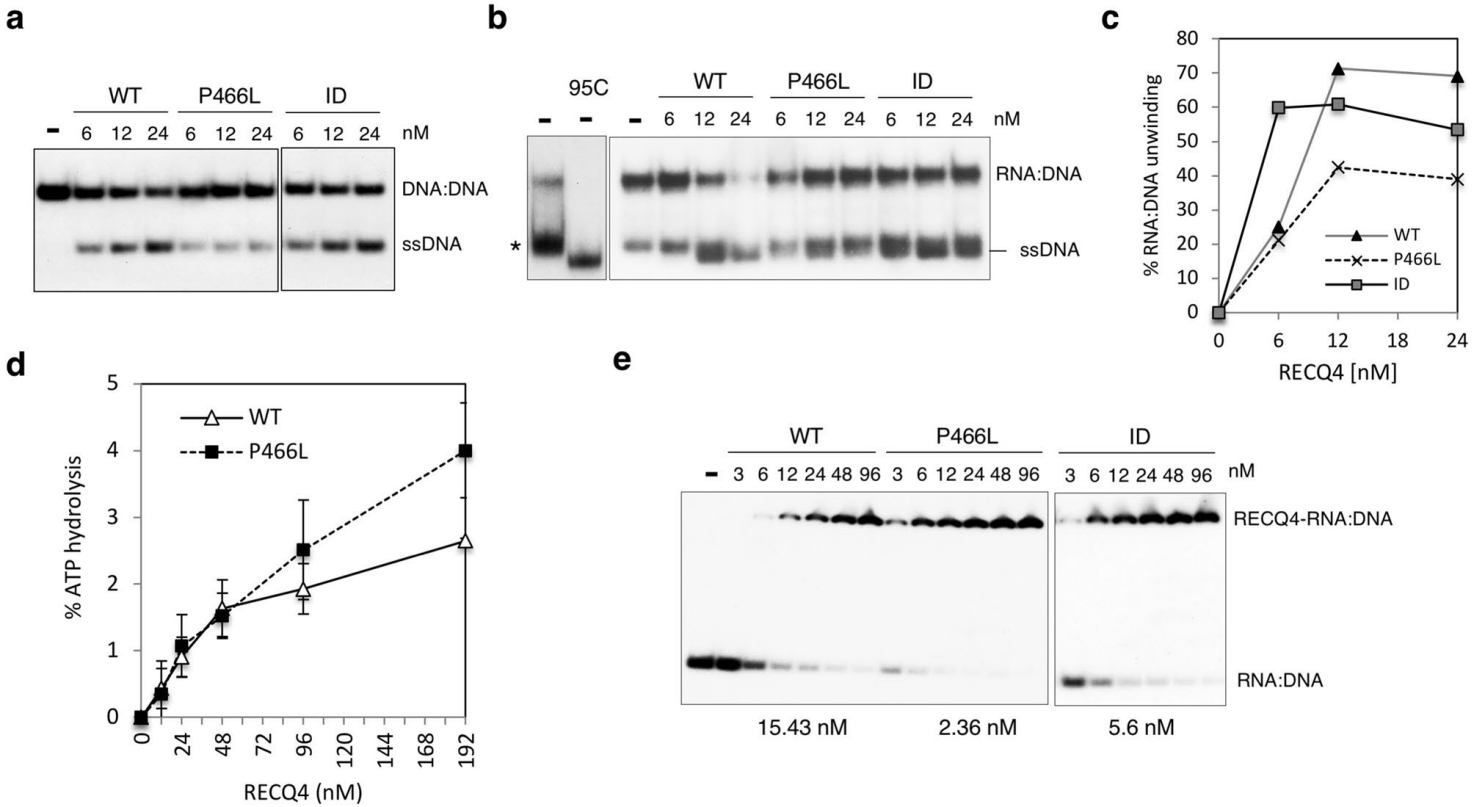
P466L mutant RECQ4 proteins exhibit reduced helicase activity on RNA:DNA hybrids. (**a**) Analysis of DNA unwinding activity of purified recombinant RECQ4 WT, P466L and ID mutant proteins using splayed-arm DNA:DNA. The X01 ssDNA strand of the splayed-arm substrate was labeled with ^32^P, and the assay was performed in the presence or absence of unlabeled oligonucleotides containing sequences identical to the ^32^P-labeled X01 strand. (**b**) Analysis of DNA unwinding activity of purified recombinant RECQ4 WT, P466L and ID mutant proteins using splayed-arm RNA:DNA substrates. The location of the dissociated ssDNA is shown in the 2nd lane. * Indicates an RNA-dependent DNA structure that is not ssDNA. (**c**) The percentages of dissociated products relative to the total amount of RNA:DNA substrates shown in (**b**) were quantified using ImageJ software. (**d**) Comparison of the ATPase activities of the RECQ4 WT and P466L mutant proteins in the presence of ssDNA. Each value represents mean ± standard deviation of 3 independent biological experiments. (**e**) Analysis of binding activity of purified recombinant RECQ4 WT, P466L and ID mutant proteins using splayed arm RNA:DNA substrates on 5% native polyacrylamide gels. Binding affinities (K_a_) indicated below the gel were quantified using ImageJ.

We next asked if RECQ4 is capable of unwinding RNA:DNA hybrid and if this activity is affected by the P466L mutation. Comparing to WT and ID mutant proteins, we found that P466L mutant protein showed reduced unwinding activity on both a splayed-arm DNA:DNA substrate (Fig. 6a) and RNA:DNA substrate (Fig. 6b,c). However, the P466L-associated decrease in unwinding activity was not due to a defect in DNA-dependent ATPase activity (Fig. 6d) or reduced binding to RNA:DNA hybrid substrate (Fig. 6e). In the contrary, P466L mutant proteins exhibited higher affinities to RNA:DNA hybrid than both RECQ4 WT and ID mutant proteins. Most likely, the enhanced annealing activity due to P466L mutation promotes reannealing of the dissociated products, leading to a reduction in unwound ssDNA observed in the helicase assays.

### P466L mutation enhances RECQ4-mtDNA association

**Figure 7:**
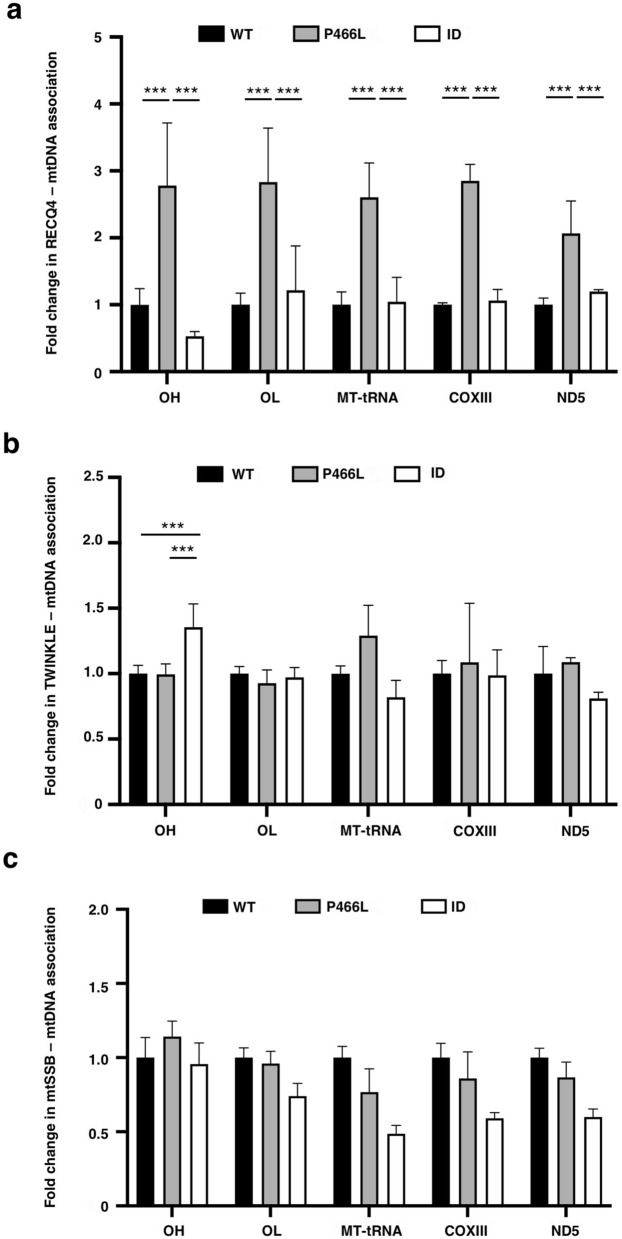
P466L mutation enhances RECQ4-mtDNA association. (**a**–**c**) Fold changes in the amount of mtDNA detected by qPCR that was co-purified with (**a**) RECQ4, (**b**) TWINKLE and (**c**) mtSSB after normalization with the input mtDNA quantified by qPCR prior to immunoprecipitation in stable RECQ4 KD HEK293 cells expressing FLAG-RECQ4 P466L or ID mutant as compared to WT expressing cells. The analysis is based on mean ± standard deviation of 3 independent biological experiments, each with 3 triplicate reactions.

The increased affinity to nucleic acids in vitro led us to ask if RECQ4 P466L mutant enhances its association with mtDNA in cells. For this, we used an anti-FLAG antibody to immunoprecipitate FLAG-tagged WT and P466L RECQ4 from WCEs prepared from HEK293 and U2OS cells treated with crosslinking agents and performed qPCR analysis. Indeed, we found that after normalization with the input mtDNA, there were significantly more P466L mutant proteins associated with all regions of the mtDNA tested in HEK293 cells compared to RECQ4 WT protein (Fig. 7a). This pattern held in U2OS cells, although to a lesser extent (Supplementary Fig. 4e). The increase in the P466L mutant proteins associated with mtDNA was unlikely contributed by the higher level of P466L mutant proteins accumulated in the mitochondria (Fig. 2g; Supplementary Fig. 3a), since the ID mutant, which also accumulated in an abundant amount in the mitochondria (Fig. 2f)^24^, did not show enhanced mtDNA association (Fig. 7a; Supplementary Fig. 4e). Given that RECQ4 interacts with TWINKLE (Fig. 2d)^24^, we also examined TWINKLE association with mtDNA and found that the level of TWINKLE proteins bound to mtDNA did not show significant change in P466L mutant cells compared to WT cells (Fig. 7b). In addition to RITOLS, asymmetric mtDNA replication can be achieved by coating the H-strand template extensively with mtSSB, a process known as strand displacement^16,18^. Our analysis showed that compared to RECQ4 WT cells, RECQ4 P466L mutant cells did not contain higher levels of mtSSB bound to mtDNA (Fig. 7c). Our results suggest that RECQ4 is involved in asymmetric mtDNA replication primarily via RITOLS but not through mtSSB binding.

Taken together, these studies reveal an increase in RECQ4 ability to promote RNA:DNA annealing and a decreased efficiency in unwinding RNA:DNA hybrids by P466L mutation. These changes in the biochemical properties of RECQ4 are consistent with the accumulation of unresolved mitochondrial RNA:DNA hybrids in P466L mutant cells.

### The clinical P466L mutation leads to a reduction in mitochondrial energy production

**Figure 8:**
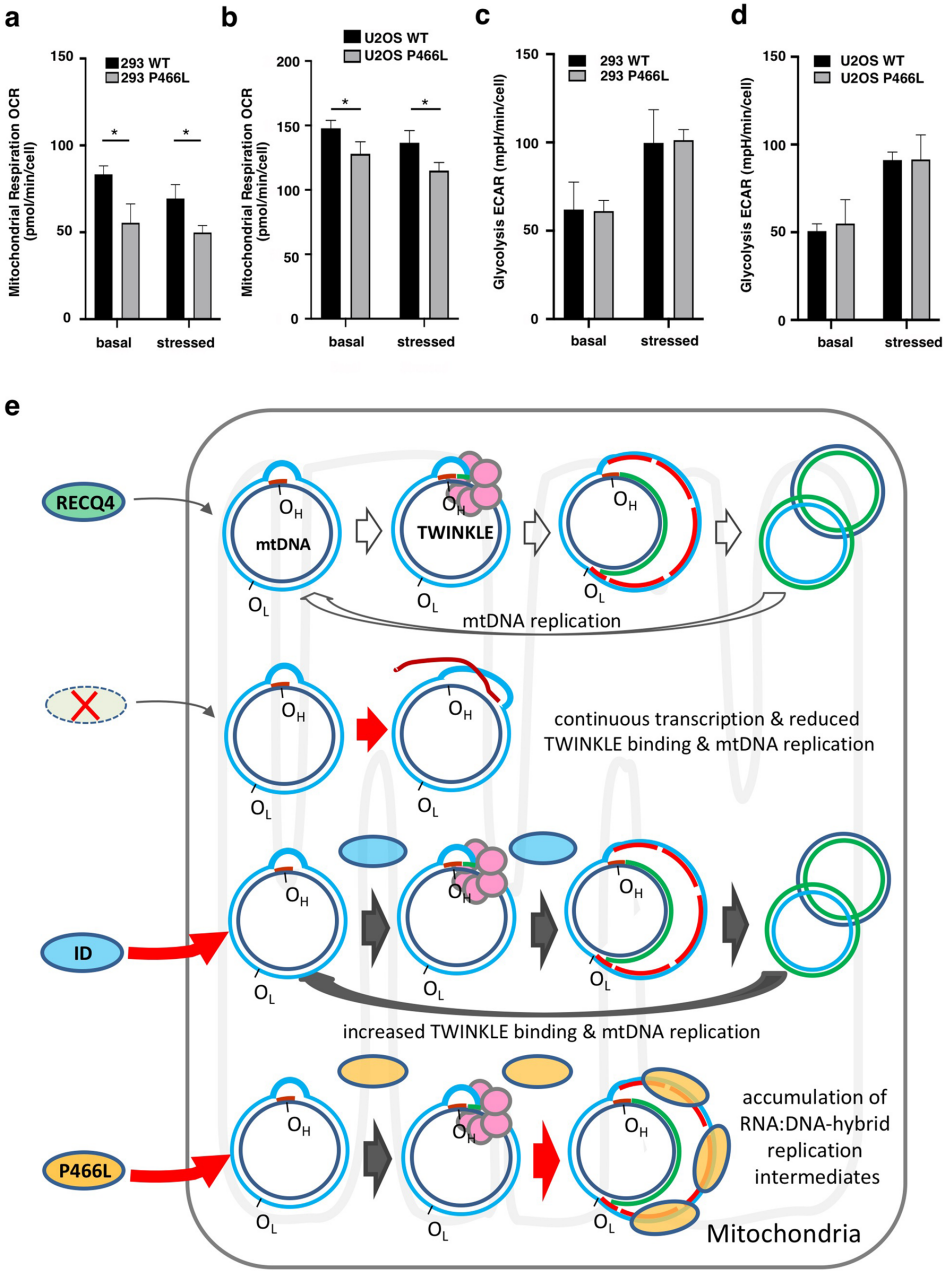
The P466L clinical mutation of RECQ4 reduces mitochondrial respiration. (**a**,**b**) Analysis of OCR measured under basal (left) and stressed (right) conditions using a Seahorse Analyzer to assess mitochondrial function in HEK293 (**a**) and U2OS (**b**) cells expressing WT or P466L mutant RECQ4. Each OCR value shown in the graph represents quantification after normalization with the input cell number. (**c**,**d**) Analysis of extracellular acidification rate (ECAR) measured under basal (left) and stressed (right) conditions using a Seahorse Analyzer to assess mitochondrial function in HEK293 (**c**) and U2OS (**d**) cells expressing WT or P466L mutant RECQ4. Each ECAR value shown in the graph represents quantification after normalization with the input cell number. (**e**) Schematic diagram of the proposed mechanism by which RECQ4 promotes mtDNA synthesis. mtDNA L- and H-strand templates are shown in light and dark blue. RNA is shown in red and the newly synthesized DNA is shown in green. See text for details.

The deregulated levels of RNA:DNA hybrids in mitochondria by the RECQ4 P466L mutation led us to ask if mitochondrial function is affected in P466L mutant cells. For this, we compared the mitochondrial function of cells expressing WT and P466L mutant RECQ4 by measuring their mitochondrial respiration OCR. We found that both HEK293 (Fig. 8a) and U2OS cells (Fig. 8b) expressing P466L mutant RECQ4 protein had a lower mitochondrial respiration OCR than WT cells, consistent with reduced mitochondrial function. On the other hand, ECAR was not affected in either HEK293 or U2OS cells expressing the P466L mutant (Fig. 8c,d), suggesting that glycolysis is not significantly increased in P466L mutant cells to compensate for reduced mitochondrial function. In conclusion, the P466L mutation of RECQ4 leads to a reduction in mitochondrial function and overall energy production. We suggest that in P466L mutant cells, mitochondrial respiratory function may have been perturbed by the accumulation of RNA:DNA hybrids or RECQ4 mutant proteins to compromise mtDNA metabolism.

## Discussion

In this paper, we demonstrated that the switch from a proline to a leucine at residue 466 is sufficient to increase nucleic acid binding affinity of RECQ4. It was reported that a cysteine-rich zinc-knuckle is present adjacent to the clinical P466L mutation sites and that a peptide consisting of this sequence is capable of binding to DNA^40^. Most likely, P466L mutation alters the secondary structure of RECQ4. The crystal structure of the human RECQ4_449-1111_ fragment revealed that residues 449–466 are unstructured, followed by a short α-helix at residues 467–475^41^. Secondary structural prediction indicates that the P466L mutation allows the α-helix to extend toward the amino terminus, converting part of the unstructured region between residues 460 and 466 into part of the α-helix. It is of great interest to confirm this structural change, which may explain the disruption of the interaction between P466L mutant RECQ4 and p32, as well as the enhanced nucleic acid binding affinity both in vitro and in cells. The higher nucleic acid binding affinity is expected to increase the level of P466L mutant RECQ4 protein bound to both mtDNA and the chromatin in the nucleus. However, like the RECQ4 ID mutant^24^, expression of RECQ4 P466L mutant protein was observed at a lower level than that of WT RECQ4 in the nucleus (Fig. 2g; Supplementary Fig. 3a), which is expected to offset the potential increase in its chromatin association.

In mammalian cells, mtDNA is duplicated via either a strand-coupled or asymmetric DNA replication mechanism, and RNA:DNA hybrid is a key replication intermediate during asymmetric DNA synthesis via RITOLS^16^. It has been reported that the RNA molecules needed for generating RNA:DNA hybrids are transcribed in an abundant amount in the mitochondria, and these preformed RNAs can then anneal to the H-strand template in a 3′–5′ direction to protect the H-strand template during mtDNA synthesis^35^. Both the formation of RNA:DNA hybrids and the timely resolution of these replication intermediates are important for efficient mtDNA synthesis. Our data provide evidence that RECQ4 promotes both the formation and resolution of RNA:DNA hybrids on mtDNA. We propose that the annealing activity of RECQ4 can contribute to RNA:DNA hybrid formation on mtDNA, while its unwinding activity can resolve RNA:DNA hybrids to allow second strand synthesis (Fig. 8e). RECQ4 can also stabilize RNA:DNA hybrids via direct binding. Increase in the annealing efficiency and reduced unwinding activity of RECQ4 by P466L mutation is consistent with the accumulation of RNA:DNA hybrids on mtDNA in the P466L mutant cells.

Our study also provides evidence that RECQ4 plays a crucial role in the initiation of mtDNA synthesis by facilitating the formation of RNA:DNA hybrid to serve as RNA primer to initiate DNA synthesis at the replication origin O_H_. Consistent with a defect in initiating mtDNA synthesis, TWINKLE associations with all mitochondrial regions tested were reduced in RECQ4-depleted cells compared to control shRNA-treated cells. On the other hand, in cells expressing RECQ4 ID mutant, which accumulates in the mitochondria and enhances mtDNA synthesis^24^, TWINKLE replicative helicase was enriched specifically at the O_H_ replication origin (Fig. 7b), providing further evidence that RECQ4 plays a role in facilitating replication initiation at O_H_ to promote mtDNA synthesis.

Our observation reveals an inverse correlation between mtDNA synthesis and mtRNA transcription, consistent with previous reports that there is a switch between mitochondrial replication initiation and transcription, as mtDNA replication at O_H_ is initiated by terminating mitochondrial transcription from LSP^15^. Indeed, RECQ4-depleted cells exhibited reduced mtDNA synthesis, and these cells contained a greater number of mitochondrial transcripts produced per mtDNA (Fig. 8e). On the other hand, the increased mtDNA replication synthesis may explain for why we also observed that RNA transcripts generated per mtDNA were reduced in the RECQ4 ID mutant cells compared to the WT RECQ4 cells (Fig. 4a)^24^. Even though RECQ4 P466L mutant cells exhibited normal replication initiation as indicated by the similar amount of TWINKLE associated with the O_H_ region as in RECQ4 WT cells, there was an increase in RNA transcripts being produced per mtDNA in these cells. Since RT-PCR using random primers is expected to amplify RNA transcripts generated from both LSP and the heavy-strand promoter (HSP), we suggest that the inability to initiate or complete the second DNA synthesis due to the persistent presence of the RNA:DNA hybrids may allow processive transcription to take place from HSP, which is also terminated early during normal mtDNA replication^15^.

Finally, although both the ID and P466L are disease-associated mutations identified in human population, patients with these mutations do not share the same disease phenotypes, such as cancer risk^30^. Elevated mtDNA content is reported to correlate with lymphoma and breast cancer risk^42^. The increase in mtDNA production specifically by the RECQ4 ID mutation but not the P466L mutation may explain for why only the ID mutation strongly associates with cancer incidence, especially lymphoma^30^.

## Materials and methods

### Plasmids

Plasmids expressing WT RECQ4 were generated by cloning *RECQ4* into a pCMV-FLAG vector, as previously described^23^. For in vitro assays, a recombinant N-terminal His-tagged and C-terminal FLAG-tagged RECQ4 (His-RECQ4-FLAG) expression plasmid was generated in a pET16b-FLAG *E. coli* expression vector, as in our previous studies^39^. All RECQ4 mutant proteins with deletions or point mutations were generated using single-primer mutagenesis of pCMV-FLAG-RECQ4 or pET16b-RECQ4-FLAG templates. The primer used for generating RECQ4 P466L mutant is listed in Supplementary Table 1. RECQ4 ID mutant constructs and inducible pLKO-tet-on–RECQ4 shRNA plasmid were generated previously^24^. To generate inducible pLKO-tet-on–TFAM shRNA plasmid, a DNA fragment containing the synthesized oligonucleotides (Supplementary Table 1) was annealed and cloned into pLKO-tet-on vector between AgeI and EcoRI sites. To induce transient KD, cells with the shRNA stably integrated into the genome were treated with 100 ng/ml DOX treatment for 48 h prior to analysis.

### Cell culture, transfection, and cell proliferation assay

HEK293 and U2OS cells were cultured in DMEM supplemented with 10% v/v FBS or Serum Plus II (Sigma-Aldrich) and streptomycin/penicillin (100 U/ml). DNA transfection was carried out using Continuum Transfection Reagent (Gemini Bio-Products) according to the manufacturer's instructions. Inducible RECQ4 KD cells were generated by stably integrating the pLKO-tet-on–RECQ4 shRNA plasmid into HEK293 cells. Single clones #1 and #2 were selected for their optimal KD efficiency after DOX treatment. Stable HEK293 and U2OS RECQ4 KD cells were generated using CRISPR technology. Two CRISPR guide RNA (gRNA) sequences, gRNA-1 (CACCGCGTGGGAGCGCGCGTTCCGA) against Exon 1 and gRNA-2 (CACCGCGCACTCTGAAGCGTACCAC) against Exon 2 were designed using online CRISPR design tools: https://tools.genome-engineering.org^43^ and cloned into pSpCas9(BB)-2A-Puro (PX459) plasmid (a gift from Dr. Feng Zhang; Addgene plasmid #48139 https://n2t.net/addgene:48139; RRID:Addgene_48139). PX459-gRNA-1 and pCAGGS-NZE-GFP (a generous gift from Dr. Jeremy M. Stark, City of Hope) were co-transfected into HEK293 cells using the Continuum Transfection Reagent. 24 h after transfection, single cells expressing GFP were selected using a BD FACSAria III cell sorter and cultured in separate wells of a 96-well plate. RECQ4 KD clones were confirmed via western blot using an anti-RECQ4 antibody. The genomic regions targeted by the gRNA were amplified using PCR with the forward primer (5′-CGG AAT TCC TGG ACG ATC GCA AGC GCG GAG GC-3′) and reverse primer (5′-CGG GAT CCG CAG GGT GCC TTT CAG ATT GGC CT-3′). The PCR products were cloned into a pCMV-FLAG vector and subjected to Sanger sequencing. To generate U2OS RECQ4 KD cells, PX459-gRNA-2 and pCAGGS-NZE-GFP were co-transfected into U2OS cells, and the RECQ4 KD cells were obtained and validated, as described for HEK293 RECQ4 KD cells. To generate cell lines stably expressing WT and mutant RECQ4, WT or mutant pCMV-FLAG-RECQ4 constructs were transfected into RECQ4 KD cells, and stable cell lines were selected using 1 mg/ml G418. Positive clones were confirmed by western blot analysis using an anti-FLAG antibody. For EtBr treatment, cells were exposed to 250 ng/ml EtBr for 7 days in medium additionally supplemented with 100 µg/ml sodium pyruvate and 50 µg/ml uridine. Crystal violet cell proliferation assays were performed as previously described^44^.

### Antibodies

Rabbit anti-FLAG (600-401-383, 1:5000) was purchased from Rockland. For immunofluorescent microscopy, mouse anti-FLAG (F1804, 1:100) was purchased from Sigma-Aldrich. Rabbit anti-MCM4 (A300-193A, 1:5000) was purchased from Bethyl Laboratories. Mouse anti-α tubulin (sc-8035, 1:1000), rabbit anti-lamin A/C (sc-20681, 1:3000) and rabbit anti-Histone H3 (sc-10809, 1:500) were purchased from Santa Cruz. Rabbit anti-VDAC1 (ab15895, 1:5000) was purchased from Abcam. Rabbit anti-p32 (6502S, 1:1000) was purchased from Cell Signaling Technology. Rabbit anti-TWINKLE (ARP36483_P050, 1:1000) and rabbit anti-TFAM (OAAN00609, 1:1000) was purchased from Aviva Systems Biology. Mouse anti-mtSSB (1:1000) was kindly provided by Dr. Valeria Tiranti (The Foundation of the Carlo Besta Neurological Institute, IRCCS, Milan, Italy).

### Cell fractionation

Cell fractionation was performed as described previously^24,37^. Briefly, the cell pellet was resuspended in mitochondrial (MT) buffer (210 mM sucrose, 70 mM mannitol, 1 mM EDTA, 1 mM EGTA, 1.5 mM MgCl_2_, 10 mM HEPES pH 7.2) containing protease and phosphatase inhibitors and incubated on ice for 30 min. After homogenization using a Dounce homogenizer (10 strokes) and centrifugation (1000×*g*, 5 min), the supernatant was collected as the cytoplasmic fraction, and the pellet was collected as intact nuclei. The pellet was further incubated on ice for 15 min in cytoplasmic buffer (10 mM Tris–HCl pH 7.5, 0.34 M sucrose, 3 mM CaCl_2_, 2 mM MgCl_2_, 0.1 mM EDTA, 1 mM DTT, 0.5% NP40). After centrifugation (2000×*g*, 2 min), nuclei were washed 2 times with 5 volumes of MT buffer and the pellet was resuspended in MT lysis buffer (20 mM HEPES pH 7.9, 1.5 mM MgCl_2_, 1 mM EDTA, 150 mM KCl, 0.1% NP40, 1 mM DTT, 10% glycerol, 0.15 U/ml benzonase, 1 × protease inhibitor, phosphatase inhibitors) and homogenized with a 21G1/2 needle, followed by incubation at 4 °C overnight. After centrifugation (18,000×*g*, 30 min), the supernatant was collected as the nuclear fraction. The cytoplasmic fraction was further centrifuged (10,000×*g*, 15 min) to pellet the mitochondria-enriched heavy membrane fraction. The supernatant was collected as the cytosolic fraction without mitochondria. The mitochondria-enriched pellet was washed with MT buffer twice, resuspended, sonicated in MT lysis buffer, incubated on ice overnight before centrifugation (18,000×*g*, 30 min), and the supernatant was collected as the mitochondrial fraction.

### Protein expression and purification

*E.coli*-expressed recombinant WT and P466L His-RECQ4-FLAG proteins were purified as previously described^39^. Plasmid-transformed *E. coli* Rosetta (DE3) pLysS cells were cultured in ampicillin (100 µg/ml) and chloramphenicol (25 µg/ml) containing Luria broth medium until the cell density (OD_600_) was 0.4. Isopropyl-β-d-thio-galactoside was then added to a final concentration of 0.1 mM, and cultures were incubated at 16 °C for 16 h. The bacteria were harvested and lysed in lysis buffer (50 mM potassium phosphate pH 8.0, 10% glycerol, 300 mM KCl, 0.5% Triton X-100, 5 mM β-mercaptoethanol, 1 × protease inhibitor, 1 mM PMSF, 0.2 mg/ml lysozyme), incubated on ice for 30 min, and sonicated at 4 °C. After centrifugation at 16,000×*g*, the sample was applied to a High-Q column (Bio-Rad), and the flow-through was collected and brought to 0.1% (w/v) in polyethyleneimine (PEI), followed by centrifugation at 16,000 × g to remove the precipitate. The supernatant was applied to a Ni–NTA column and washed sequentially with 1 × buffer B (50 mM potassium phosphate pH 6.0, 10% glycerol, 500 mM KCL, 0.5% Triton X-100, 5 mM β-mercaptoethanol) containing 5 mM imidazole and 1 × buffer B containing 50 mM imidazole, and the His-tagged proteins were eluted with buffer B containing 1 M imidazole. After dialysis against buffer D (50 mM Tris–HCl pH 7.6, 10% glycerol, 500 mM KCl, 0.2% Triton X-100, 1 mM EDTA), the eluate was incubated overnight with anti-FLAG M2 beads (Sigma-Aldrich) at 4 °C. The M2-bound proteins were washed with buffer E (50 mM Tris–HCl pH 7.6, 10% glycerol, 500 mM KCl, 0.2% Triton X-100, 1 mM EDTA, 1 mM DTT), eluted with 3X FLAG peptide elution buffer (50 mM Tris–HCl pH 7.4, 150 mM NaCl, 100 µg/ml 3X FLAG peptide [Sigma-Aldrich]), and dialysed against buffer F (50 mM Tris–HCl, pH8.0, 10% glycerol, 500 mM KCl, 1 mM EDTA, 1 mM DTT). The concentrations of the purified proteins were estimated relative to the Coomassie blue staining signals of BSA standards on SDS–polyacrylamide gels.

### DRIP and protein-mtDNA association

ACTB exon 3 DRIP was performed as previously described^38,45^. For mtDNA DRIP analysis, cells were harvested and fractionated, and the mitochondrial fraction was lysed in SDS/Proteinase K buffer (50 mM Tris pH 8.0, 10 mM EDTA, 0.5% SDS, 300 μg/ml proteinase K) at 37 °C overnight. After phenol/chloroform extraction and ethanol precipitation, nucleic acids were fragmented using restriction enzymes EcoRV and SacI with or without RNase H at 37 °C overnight. Fragmented nucleic acids were purified using phenol/chloroform extraction and ethanol precipitation, then 2 µg per sample was immunoprecipitated with 2.5 μg of anti-S9.6 antibody and 25 μl protein A agarose in binding buffer (10 mM NaPO_4_ pH 7.0, 140 mM NaCl, 0.05% Triton X- 100) at 4 °C for 1 h, followed by extensive washing in binding buffer. The bound nucleic acids were eluted with SDS/Proteinase K buffer at 50 °C for 1 h. For protein-mtDNA association analysis, cells were fixed with 2 mM dithiobis (succinimidyl propionate) for 45 min (FLAG-RECQ4) or with 1% formaldehyde for 10 min (mtSSB and TWINKLE) at room temperature, and the reaction was stopped by the addition of 125 mM glycine. After washing three times with PBS, the cells were lysed in ChIP nuclei lysis buffer (50 mM Tris–HCl pH 8.0, 10 mM EDTA, 1% SDS, 1 × protease inhibitor, phosphatase inhibitors), and chromatin was sheared to approximately 100–500 bp by sonication. The fragmented chromatin was diluted 5 times in ChIP dilution buffer (20 mM Tris–HCl pH 8.0, 157 mM NaCl, 0.01% SDS, 1.1% Triton X-100, 1.1 mM EDTA, 1 × protease inhibitor, phosphatase inhibitors) and incubated overnight with antibodies. Salmon sperm DNA-blocked protein A beads were added to pull down the antibody/protein/DNA complexes with rotation for 1 h at 4 °C. The beads were washed sequentially twice with low salt buffer (20 mM Tris–HCl pH 8.0, 150 mM NaCl, 0.1% SDS, 1% Triton X-100, 2 mM EDTA), high salt buffer (20 mM Tris–HCl pH 8.0, 500 mM NaCl, 0.1% SDS, 1% Triton X-100, 2 mM EDTA), LiCl buffer (20 mM Tris–HCl pH 8.0, 0.5 M LiCl, 1% NP-40, 1 mM EDTA, 1% deoxycholate), and TE buffer (10 mM Tris–HCl pH 8.0, 1 mM EDTA). The antibody/protein/DNA complexes were eluted with 300 µl elution buffer (0.1 M sodium bicarbonate, 1.0% SDS) at room temperature for 15 min, reverse cross-linked by adding 20 μl of 5 M NaCl, and incubated at 65 °C overnight, followed by treatment with Proteinase K at 45 °C for 2 h and purification by phenol/chloroform extraction and ethanol precipitation. For both DRIP and protein-mtDNA association analyses, the input nucleic acids and the purified nucleic acids were both subjected to qPCR using primers against O_H_, O_L_, COXIII, ND5 and mt-tRNA (Supplementary Table 2). qPCR was performed using the ABI 7500 Fast Real-Time PCR system and SYBR Green. Enrichment was calculated using the comparative Ct method.

### mtDNA level, RT and qPCR

mtDNA measurement was performed as previously described^24^. For RT, total RNA was isolated using the Quick-RNA MiniPrep kit (Zymo Research). Quality and quantity of RNA was analyzed by NanoDrop ND1000 spectrophotometer. Isolated cellular RNA was used for cDNA synthesis using a High-Capacity RNA-to-cDNA Kit (Applied Biosystems). qPCR was performed in triplicate using SYBR Green Master Mix (Applied Biosystems) on a Light Cycler 7500 Fast (Applied Biosystems).

### Helicase, ATPase, DNA annealing, and DNA binding assays

Helicase, ATPase, DNA annealing, and DNA binding assays were performed as previously described^39^. The radiolabeled splayed-arm DNA:DNA substrate was prepared by labeling X01 oligonucleotides (5′- GAC GCT GCC GAA TTC TAC CAG TGC CTT GCT AGG ACA TCT TTG CCC ACC TGC AGG TTC ACC C-3′) with [γ-^32^P] ATP prior to annealing to unlabeled X04 oligonucleotides (5′- ATC GAT AGT CGG ATC CTC TAG ACA GCT CCA TGT AGC AAG GCA CTG GTA GAA TTC GGC AGC GT-3′). The annealed products were gel-purified prior to analysis. For splayed-arm RNA:DNA substrates, ^32^P-labeled X01 oligonucleotides were annealed to unlabeled X04 RNA oligonucleotides (5′- AUC GAU AGU CGG AUC CUC UAG ACA GCU CCA UGU AGC AAG GCA CUG GUA GAA UUC GGC AGC GU-3′). Helicase assays were performed by incubating the purified WT or mutant RECQ4 proteins with ^32^P-labeled substrates in helicase buffer (30 mM Tris–HCl pH 7.5, 5 mM MgCl_2_, 5 mM ATP, 1 mM DTT, 100 μg/ml BSA, 10% glycerol) at 37 °C for 30 min. The reactions were stopped by adding stop buffer (30 mM Tris–HCl pH 7.5, 5 mM MgCl_2_, 5 mM ATP, 1 mM DTT, 100 µg/ml BSA, 10% glycerol) and 2.5 ng unlabeled X01 at 37 °C for 15 min. The reactions were deproteinized and analyzed on polyacrylamide gels. Annealing reactions were carried out by incubating WT or mutant RECQ4 proteins with 10 pg of ^32^P-labeled X01 and 25 pg of unlabeled X04 or X04 RNA in annealing buffer (30 mM Tris pH 7.5, 5 mM MgCl_2_, 50 mM KCl, 1 mM DTT, 100 μg/ml BSA, 10% glycerol) at 37 °C for 5 min, followed by incubation with stop buffer at 37 °C for 15 min. DNA binding assays were performed by incubating WT or mutant RECQ4 proteins with 4 pg of ^32^P-labeled DNA:DNA or RNA:DNA in DNA binding buffer (30 mM HEPES pH 7.5, 1 mM DTT, 100 µg/ml BSA) on ice for 15 min. The reactions were cross-linked with 1% glutaraldehyde at 37 °C for 15 min, and the protein-DNA complexes were analyzed on 5% native polyacrylamide gels. For ATPase activity assays, purified recombinant WT or P466L RECQ4 proteins were incubated with 1 μg ssDNA and 2.5 × 10^2^ μCi [γ-^32^P] ATP in a 10 μl reaction containing ATPase buffer (30 mM Tris pH7.5, 10% glycerol, 5 mM MgCl_2_, 50 μM cold ATP, 1 mM DTT, 100 μg/ml BSA) for 1 h at 37 °C. The reactions were stopped by adding 0.5 μl of 0.5 M EDTA, then separated on a PEI-cellulose thin-layer chromatography plate using a solution of 0.8 M LiCl and 1 M formic acid, and incubated in a moist chamber for 1.5 h. The percentage of ATPase hydrolysis was quantified using ImageJ software.

### Seahorse mitochondrial stress test

Seahorse XF Cell Mitochondrial Stress Test (Agilent) was carried out as recommended based on the manufacturer’s protocol. To prepare cell samples for analysis, 10^5^ cells per well were seeded for overnight incubation in XF96 tissue culture plates supplemented with 5% CO_2_. Cells were then washed twice with 200 µL of XF base medium (pre-warmed) supplemented with 2 mM glutamine, 25 mM glucose and 1 mM sodium pyruvate pH 7.4, followed by 1 h incubation in 150 µl of XF base medium plus 25 µl growth medium (175 µl final) at 37 °C without CO_2_ prior to analysis. To perform the Seahorse XF Cell Mitochondrial Stress Test, the following pre-warmed reagents in the amount of 25 µl each were loaded into injector ports of the sensor cartridge calibrated on the XF96 analyzer (Seahorse Bioscience, Billerica, MA, USA): oligomycin to port A (0.8 µM final), FCCP to port B (2.5 µM), and antimycin A to port C (1 µM final). OCR was first measured under basal conditions, and additional measurements were taken after the sequential addition of oligomycin, FCCP, and antimycin A. The measurements were normalized to cell numbers based on MTT cell proliferation assays.

### Immunoprecipitation

Cells were harvested and disrupted in 0.5% Triton X-100 lysis buffer (50 mM Tris–HCl pH7.4, 150 mM NaCl, 1 mM EDTA, 0.5% Triton X-100, 10% glycerol, 1 × protease inhibitor). Soluble cell fractions were isolated by centrifugation for 10 min at 16,000 × g, and immunopurification of FLAG-tagged protein complexes from the cell lysates was performed as previously described^38,46^. Representative immunoblots from a minimum of three independent experiments are shown.

### Immunofluorescence assay

To detect RECQ4 localization, coverslip-cultured FLAG-RECQ4 mutant HEK293 cells were treated with 100 nM MitoTacker (Red CMXRos #9082, Cell Signaling) at 37 °C for 30 min and then fixed with 4% paraformaldehyde at RT for 20 min, followed by permeabilized with 0.1% Triton X-100 at RT for 5 min. The coverslips were incubated with monoclonal anti-FLAG antibody at 37 °C for 1 h. After washing with PBS, the coverslips were incubated with Alexa Fluor Plus 488 (Invitrogen) at 37 °C for 1 h and the cells were covered with mounting medium (SlowFade Gold Antifade Mountant, Invitrogen). The staining patterns were observed under a Zeiss LSM 880 with Airyscan confocal microscope.

### Statistics and Reproducibility

A minimum of three independent biological experiments were performed for each figure. For graphs, each data point represents mean of 3 independent biological experiments, each with 3 technical replicates, ± standard deviation. Protein-mtDNA association was quantified as the percentage of a specific sequence in the input mtDNA being pulled down using an antibody against protein of interest minus the percentage from the quantification using protein A beads without antibody. Similarly, DRIP was quantified as the percentage of the input mtDNA being immunopurified using S9.6 antibody minus the quantification from the RNase H treatment. Both the input and immunopurified DNA were quantified by qPCR. The normalization of the immunopurified mtDNA sequence to input mtDNA ensures that each value takes into account the variation in mtDNA levels among different cell lines. p values were calculated using two-tailed student’s t-tests for statistically significant differences. * indicates *p* value < 0.05, ** indicates *p* value < 0.01; and *** indicates *p* value < 0.005. All in vitro analyses using purified recombinant proteins were performed at least three times using 2 different sets of purified proteins.

## Supplementary information

Supplementary Information.
