# Supplementary material for: Pathogenic mutations reveal a role of RECQ4 in mitochondrial RNA:DNA hybrid formation and resolution
Source: Sci Rep. 2020 Oct 12;10:17033. doi: 10.1038/s41598-020-74095-9 (PMC7552406; doi:10.1038/s41598-020-74095-9)
Supplement: Supplementary file 1 — Supplementary Information. [file 41598_2020_74095_MOESM1_ESM.pdf]

Pathogenic mutations reveal a role of RECQ4 in mitochondrial RNA:DNA hybrid formation and resolution

(Supplementary Information)

Chou-Wei Chang[1], Xiaohua Xu[1], Min Li[1], Di Xin[1], Lin Ding[2], Ya-Ting Wang[3], and Yilun Liu[1,4]

[1]Department of Cancer Genetics and Epigenetics, Beckman Research Institute,

City of Hope, Duarte, CA 91010-3000

[2]J. Craig Venter Institute, San Diego, CA 92037

[3] Memorial Sloan Kettering, New York, NY 10065

[4]To whom correspondence should be addressed: yiliu@coh.org

Keywords: RECQ4, mitochondrial DNA replication, RNA:DNA hybrid

Running Title: Mitochondrial function of RECQ4

Supplementary Figure Legends

Supplementary Fig. 1. RECQ4 is important for mitochondrial function.

(a) Analysis of extracellular acidification rate (ECAR) measured under basal (left) and stressed (right) conditions using a Seahorse Analyzer to assess mitochondrial function in control and RECQ4-depleted HEK293 cells. Each ECAR value shown in the graph represents quantification after normalization with the input cell number.

(b) Fold changes in mtDNA:genomic DNA (gDNA) ratio in RECQ4-depleted HEK293 cells as compared to control shRNA cells treated with (right) or without (left) ethidium bromide (EtBr) for 7 days. *** indicates p value < 0.005.

(c) Fold changes in the rates of mtDNA recovery quantified in DOX-induced RECQ4-depleted HEK293 cells relative to control shRNA-treated cells 7-day after release from the treatment with EtBr.

Supplementary Fig. 2. Generation of RECQ4 CRISPR KO cells.

(a-c) Sequencing analysis of Exon 1 targeted by CRISPR gRNA of the RECQ4 alleles isolated from HEK293 WT (a) and RECQ4 KD (b-c) cells, the latter of which contain 1 nucleotide deletion in allele 1 (b) and 4 nucleotide deletion in allele 2 (c).

(d) Western blot analysis of RECQ4 in WCEs prepared from U2OS WT or RECQ4 CRISPR KO cells. Tubulin is used as a loading control.

(e-h) Sequencing analysis Exon 3 targeted by CRISPR gRNA of the RECQ4 alleles isolated from U2OS WT (e) and stable RECQ4 KD (f-h) cells.

Supplementary Fig. 3. RECQ4 P466L mutation hinder mtDNA synthesis

(a) Representative western blot analysis of RECQ4 WT and P466L mutant in WCEs and cytosolic (Cyt), mitochondrial (MT), and nuclear (Nuc) fractions prepared from RECQ4 KD U2OS cells expressing the corresponding FLAG-RECQ4 proteins. Tubulin, VDAC1, and lamin A/C are loading and fractionation controls for Cyt, MT, and Nuc fractions, respectively.

(b) Fold changes in mtDNA:gDNA ratio in RECQ4 KD U2OS cells expressing P466L or ID mutant RECQ4 as compared to cells expressing WT RECQ4. *** indicates p value < 0.005.

(c) Fold changes in mtDNA:gDNA in stable RECQ4 KD HEK293 cells expressing P466L or ID mutant RECQ4 as compared to cells expressing WT RECQ4 treated with (right) or without (left) ethidium bromide (EtBr). *** indicates p value < 0.005. N.S. indicates not significant.

(d) Fold changes in the rates of mtDNA recovery in RECQ4 KD HEK293 cells expressing P466L mutant 7 days after release from the treatment with EtBr as compared to those expressing WT RECQ4.

Supplementary Fig. 4. Effect of RECQ4 P466L mutation on mitochondrial RNA:DNA hybrids and RECQ4-mtDNA association in U2OS cells

(a-d) Fold changes in RNA:DNA hybrid frequency at (a) O_H (OH), (b) O_L (OL), (c) mt-tRNA and (d) COXIII gene loci on mtDNA in stable RECQ4 KD U2OS cells expressing FLAG-RECQ4 P466L or ID mutant constructs as compared to cells expressing WT RECQ4. RNA:DNA hybrid frequency was measured by DRIP.

(e) Fold changes in the amount of mtDNA detected by qPCR that was co-purified with RECQ4 after normalized with the mtDNA input prior to immunoprecipitation in stable RECQ4 KD

U2OS cells expressing FLAG-RECQ4 P466L or ID mutant as compared to WT expressing cells.

Supplementary Fig. 5. Original scanned western blot gel images

Original scanned images for (a-d) Fig. 1a, (e) Fig. 1b, (f-i) Fig. 2b, (j-p) Fig. 2d and (q) Fig. 2f. For all the images shown in this figure, red dashed rectangles indicate the locations of the cropped images.

Supplementary Fig. 6. Original scanned western blot gel images

Original scanned images for (a-d) Fig. 2g, (e) Fig. 4b, (f-g) Supplementary Fig. 2d and (h-j) Supplementary Fig. 3a. For all the images shown in this figure, red dashed rectangles indicate the locations of the cropped images.

Supplementary Table 1: Primers for mutagenesis and shRNA

ID	5'-CCAGTGTCCCCGGCCAGAGACGCCGGCTGAGG-3'
P466L	5'-GCAGTTGGCAGAGACGCTGGCTGAGGTGTTCCAGG-3'
shTFAM-1 Forward	5'- CCGG GTAAGTTCTTACCTTCGATTT CTCGAG AAATCGAAGGTAAGAACTTAC TTTTT-3'
shTFAM-1 Reverse	5'- AATTAAAAA GTAAGTTCTTACCTTCGATTT CTCGAG AAATCGAAGGTAAGAACTTAC-3'

Supplementary Table 2: qPCR Primers

	Forward	Reverse
mt-tRNA	5'-CACCCAAGAACAGGGTTTGT-3'	5'-TGGCCATGGGTATGTTGTTA-3'
mt-O_L	5'-AAGCCCCGGCAGGTTTGAAG-3'	5'-TGGGGGTGAGGTAAAATGGC-3'
mt-O_H	5'-GTGGCTTTGGAGTTGCAGTT-3'	5'-GAAGCAGATTTGGGTACCAC-3'
β-globin	5'-TGTGTTCACGACTGACATCACCG-3'	5'-GCTGGGCTTCTGTTGCAGTAGGG-3'
ACTB EX3	5'-GCTCAGGGCTTCTTGTCCTT-3'	5'-TCGATGGGGTACTTCAGGGT-3'
MT-COXI	5'-CTGCTATAGTGGAGGCCGGA-3'	5'-GGGTGGGAGTAGTTCCCTGC-3'
MT-COXIII	5'-CCAATGATGGCGCGATG-3'	5'-CTTTTTGGACAGGTGGTGTGTG-3'
MT-ND1	5'-ACGCCATAAAACTCTTCACCAAAG-3'	5'-TAGTAGAAGAGCGATGGTGAGAGCTA-3'
MT-ND5	5'- AGTTACAATCGGCATCAACCAA-3'	5'- CCCGGAGCACATAAATAGTATGG-3'

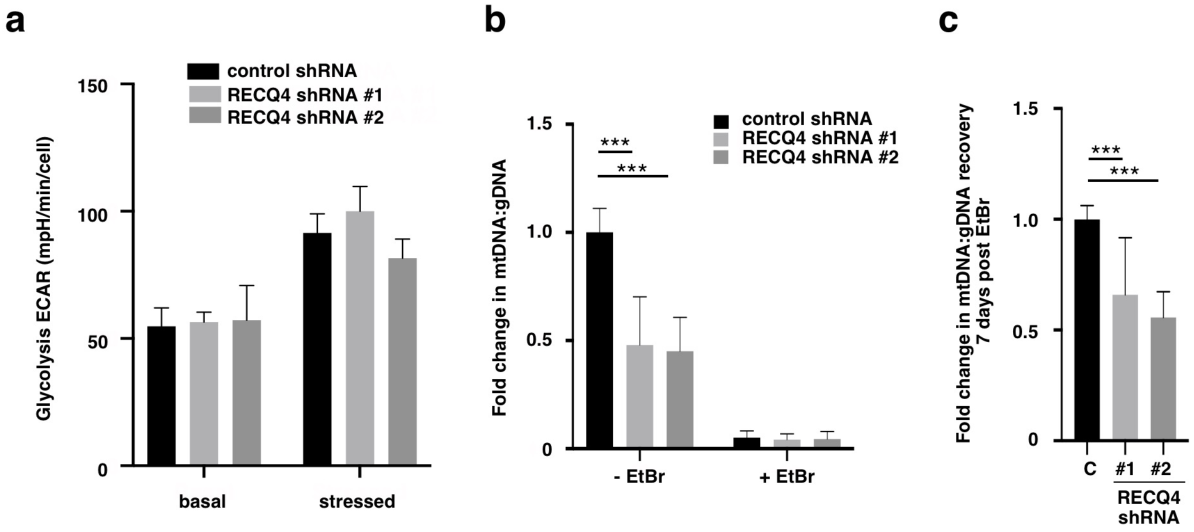

Chang_Supplementary_Fig_1

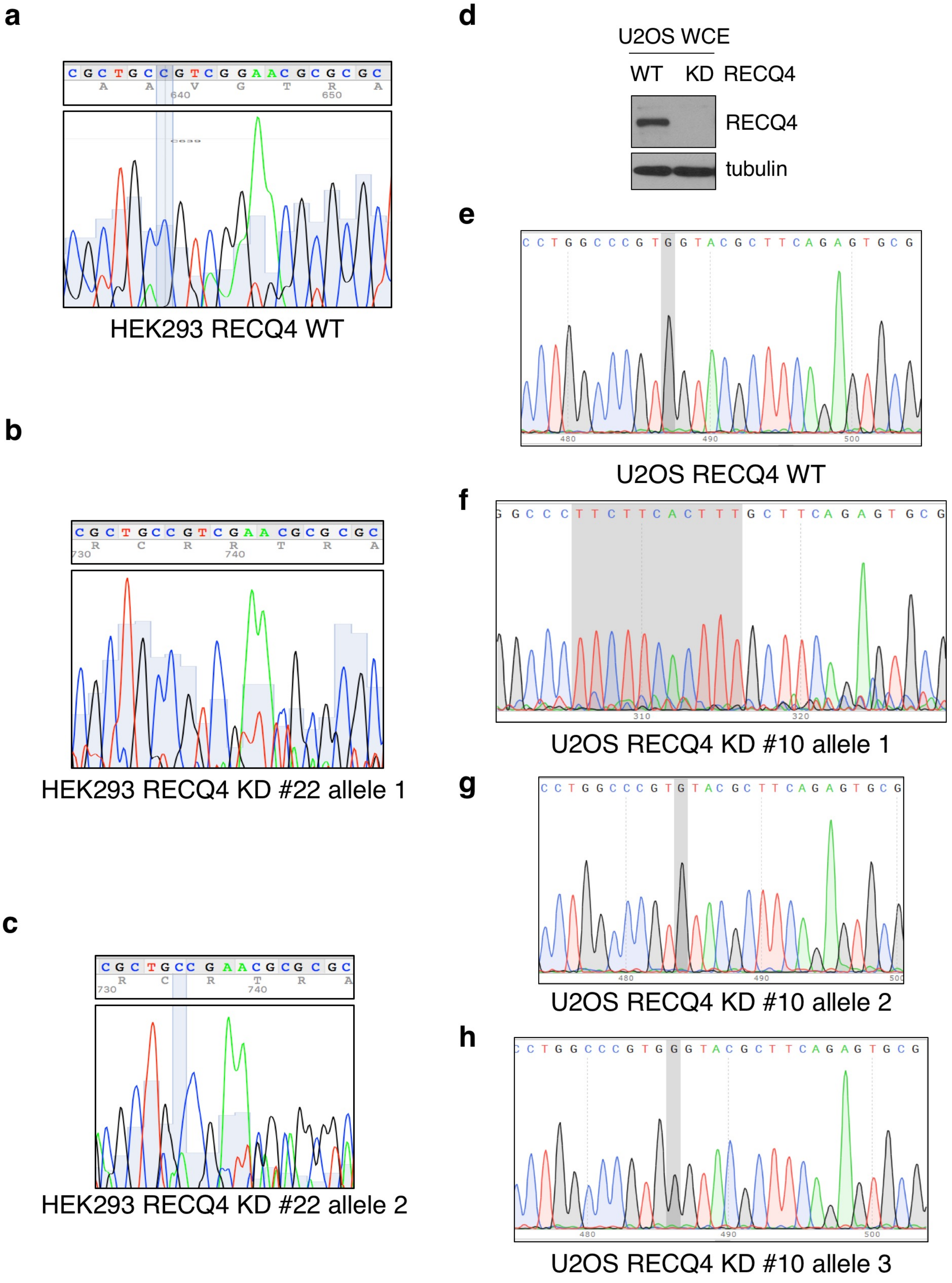

Chang_Supplementary_Fig_2

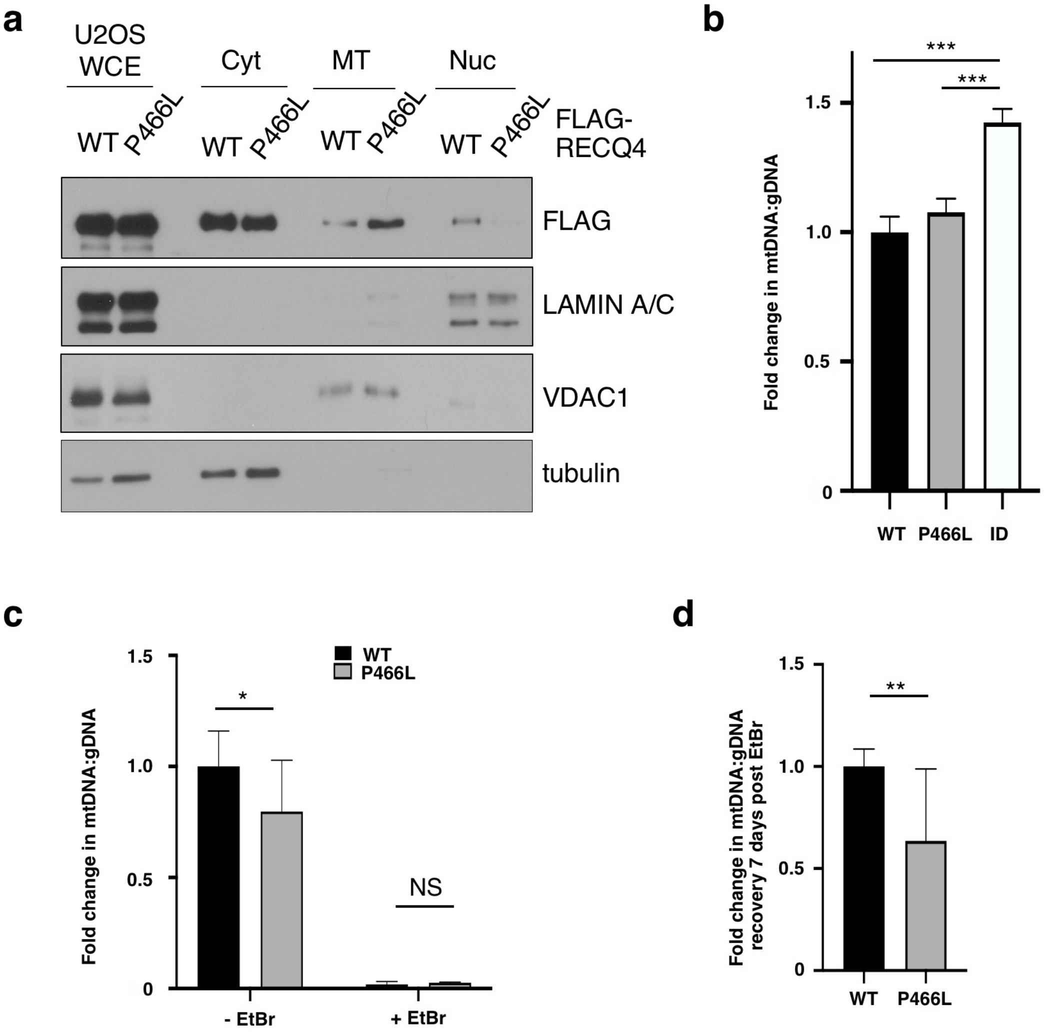

Chang_Supplementary_Fig_3

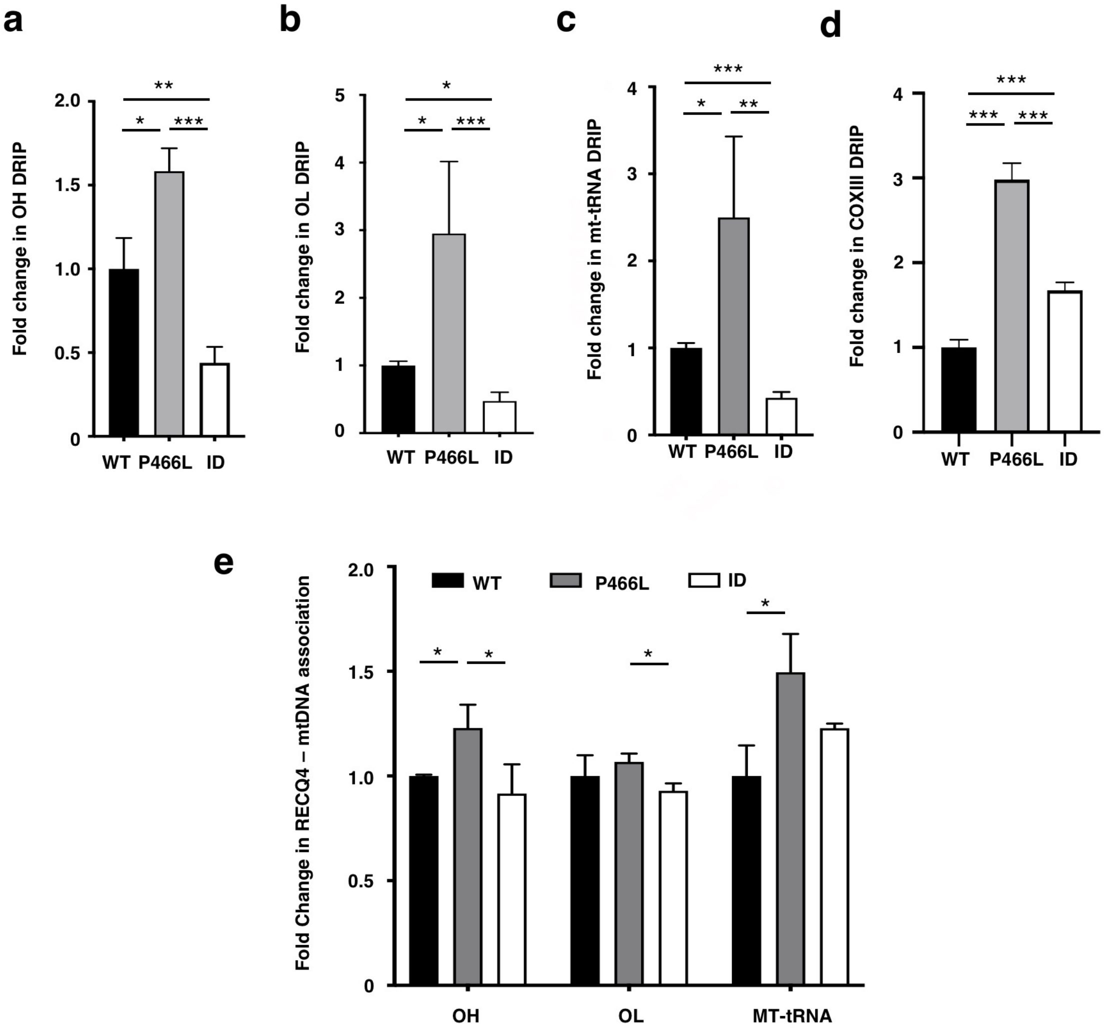

Chang_Supplementary_Fig_4

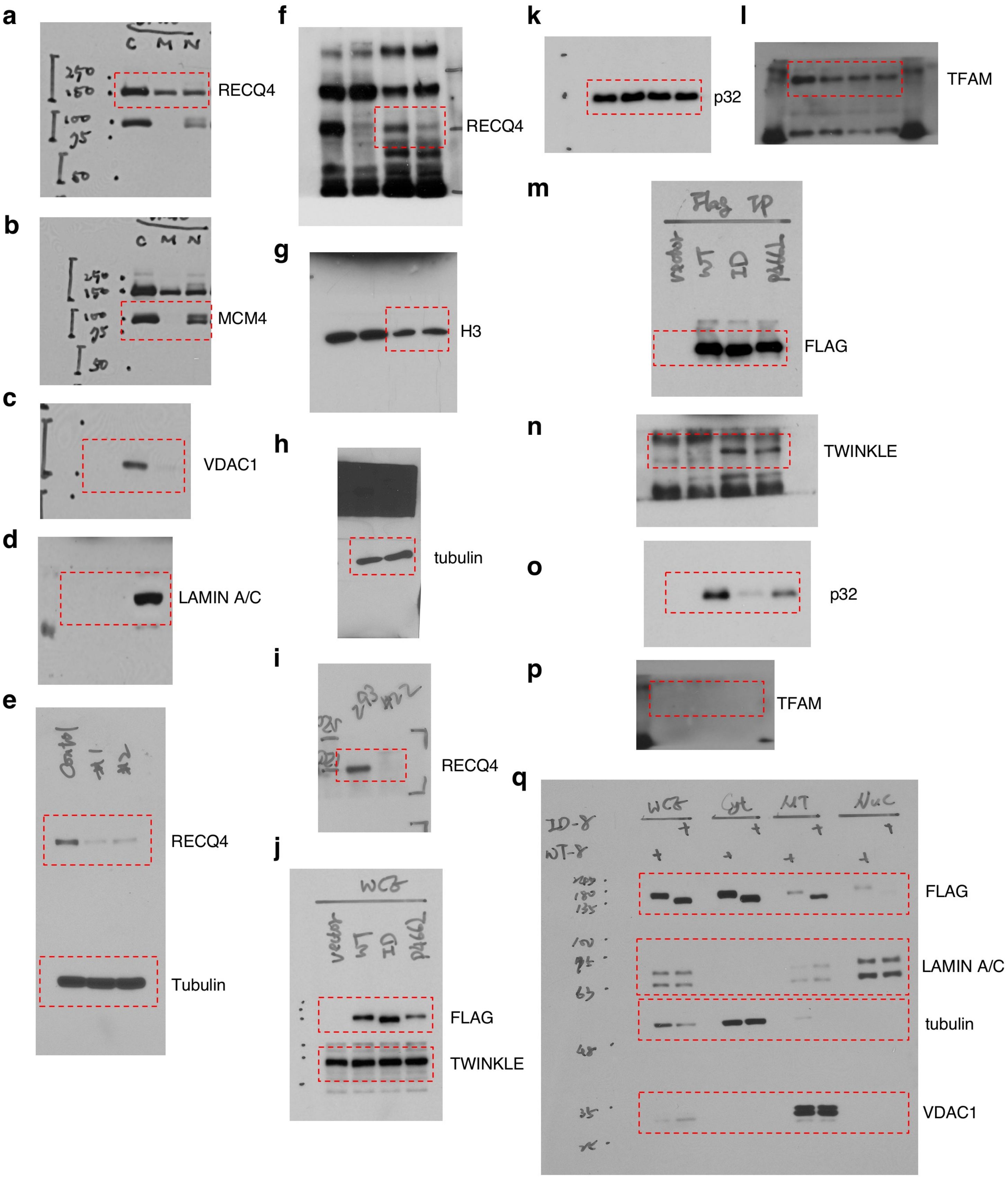

Chang_Supplementary_Fig_5

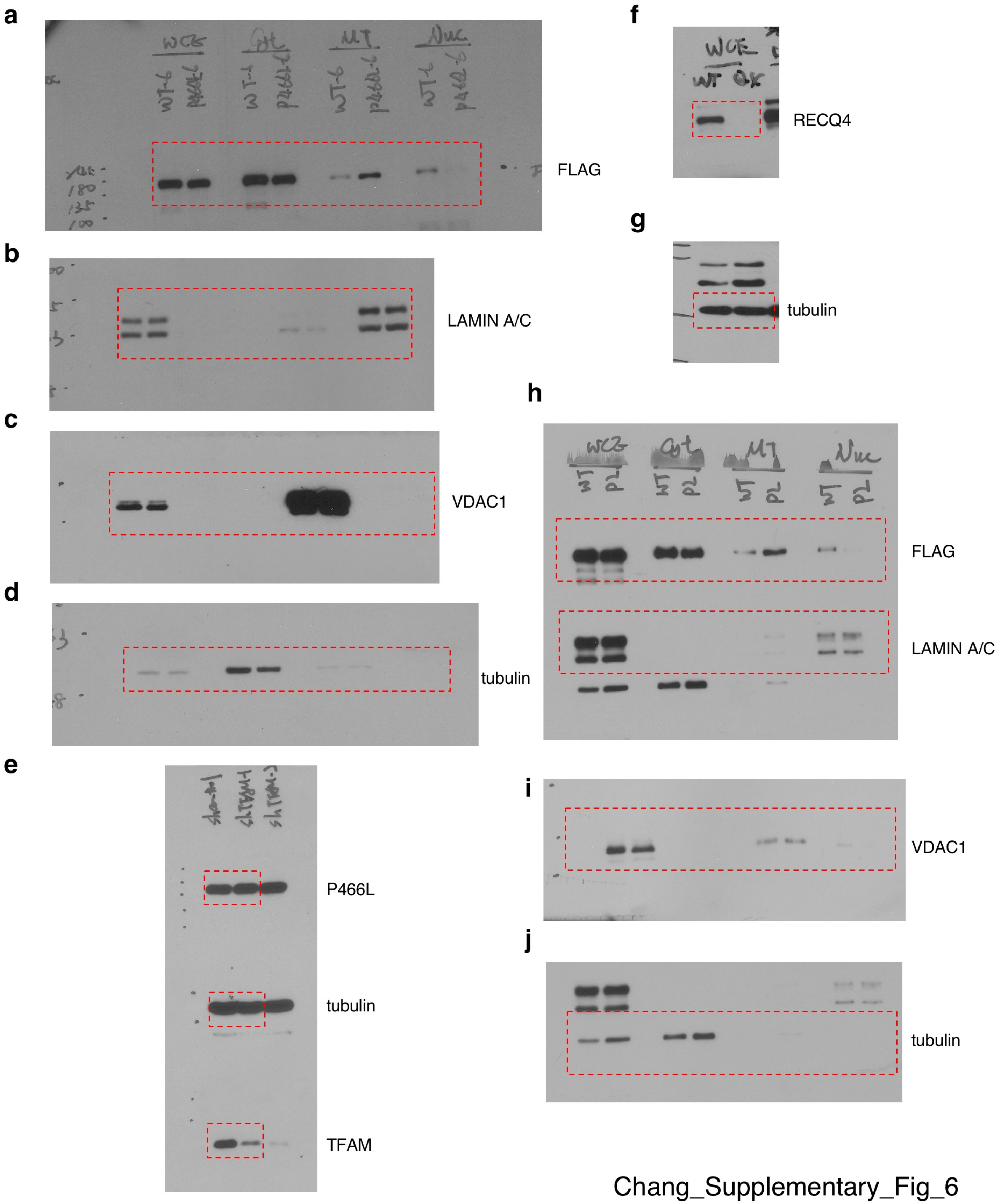

Chang_Supplementary_Fig_6
